# Supplementary material for: Cranial bone histology of Metoposaurus krasiejowensis (Amphibia, Temnospondyli) from the Late Triassic of Poland
Source: PeerJ. 2016 Nov 8;4:e2685. doi: 10.7717/peerj.2685 (PMC5103832; doi:10.7717/peerj.2685)
Supplement: Supplemental Information 1 [file peerj-04-2685-s001.pdf]

The detailed histological description of the skull bones of *Metoposaurus* from the Late Triassic of Poland

Supplementary material to:

Cranial bone histology of *Metoposaurus krasiejowensis* (Amphibia, Temnospondyli) from the Late Triassic of Poland

Kamil Gruntmejer[1,2,*], Dorota Konietzko-Meier[1,2,3], Adam Bodzioch[1,2]

[1] Opole University, Department of Biosystematics, Laboratory of Palaeobiology, Oleska 22, 45-052 Opole, Poland

[2] Opole University, European Centre of Palaeontology, Oleska 48, 45-052 Opole, Poland

[3] University of Bonn, Steinmann Institute, Nussallee 8, 53115 Bonn, Germany

*gruntmejerkamil@gmail.com

Table of contents:

Histology of the dermal bones of skull roof…………………………...…………………...3

Premaxilla……………………………………………………………………......3

Maxilla…………………………………………………………………….....5

Nasal……………………………………………………………………………7

Lacrimal………………………………………………………………………..9

Prefrontal……………………………………………………………………...12

Jugal & Postorbital……………………………………………………………14

Postfrontal…………………………………………………………………….17

Frontal………………………………………………………………………...20

Parietal……………………………………………………………………………...22

Supratemporal………………………………………………………………...25

Squamosal 1…………………………………………………………..………27

Squamosal 2………………………………………………………..……30

Postparietal…………………………………………………………………32

Tabular……………………………………………………………………..35

Quadratojugal……………………………………………………………37

Histology of the dermal bones of palate…………………………...……………………40

Vomer……………………………………………………………………....40

Parasphenoid…………………………………………………………….43

Pterygoid……………………………………………………………...45

Quadrate…………………………………………………………………………....46

Exoccipital……………………………………………………………………….…...48

Histology of dermal bones of skull roof

Premaxilla

The sample of the right premaxilla was analyzed histologically (Fig. 1). The thickness of the alary process is from 3 mm to 5 mm, and the ratio between E:M:I is 1:1.1:0.9.

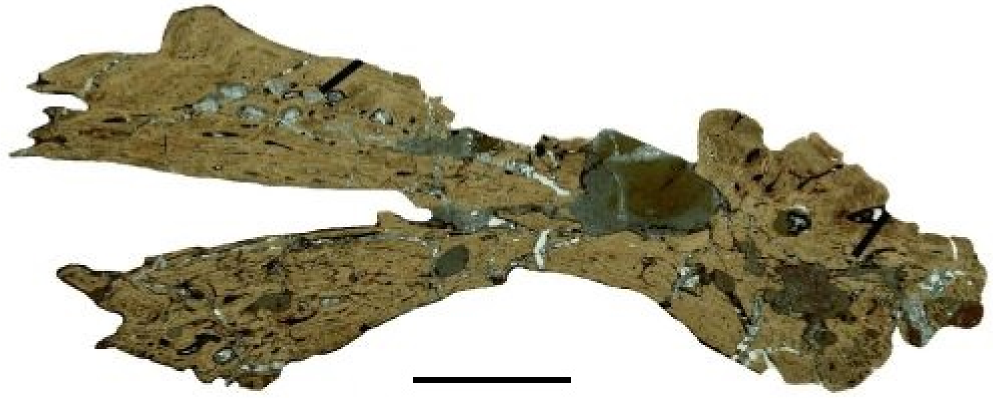

Fig. 1. Histological section of the right premaxilla. Scale bar equals 5 mm.

External cortex - The parallel-fibered bone is poorly vascularized. Well mineralized Sharpey’s fibers are relatively short but very numerous and occur only in the outermost part of the bone (Fig. 2A). The growth marks are not visible.

Middle region - The external cortex transits to cancellous middle region characterized by relatively large erosion cavities, up to 2000 µm in diameter (Fig. 2B, C). Middle region is well vascularized (Fig. 2D). In some parts of the middle region the relatively high concentration of secondary osteons is visible (Fig. 2E).

The **internal cortex** is poorly vascularized but higher than the external cortex, and consists of parallel-fibered bone (Fig. 2F).

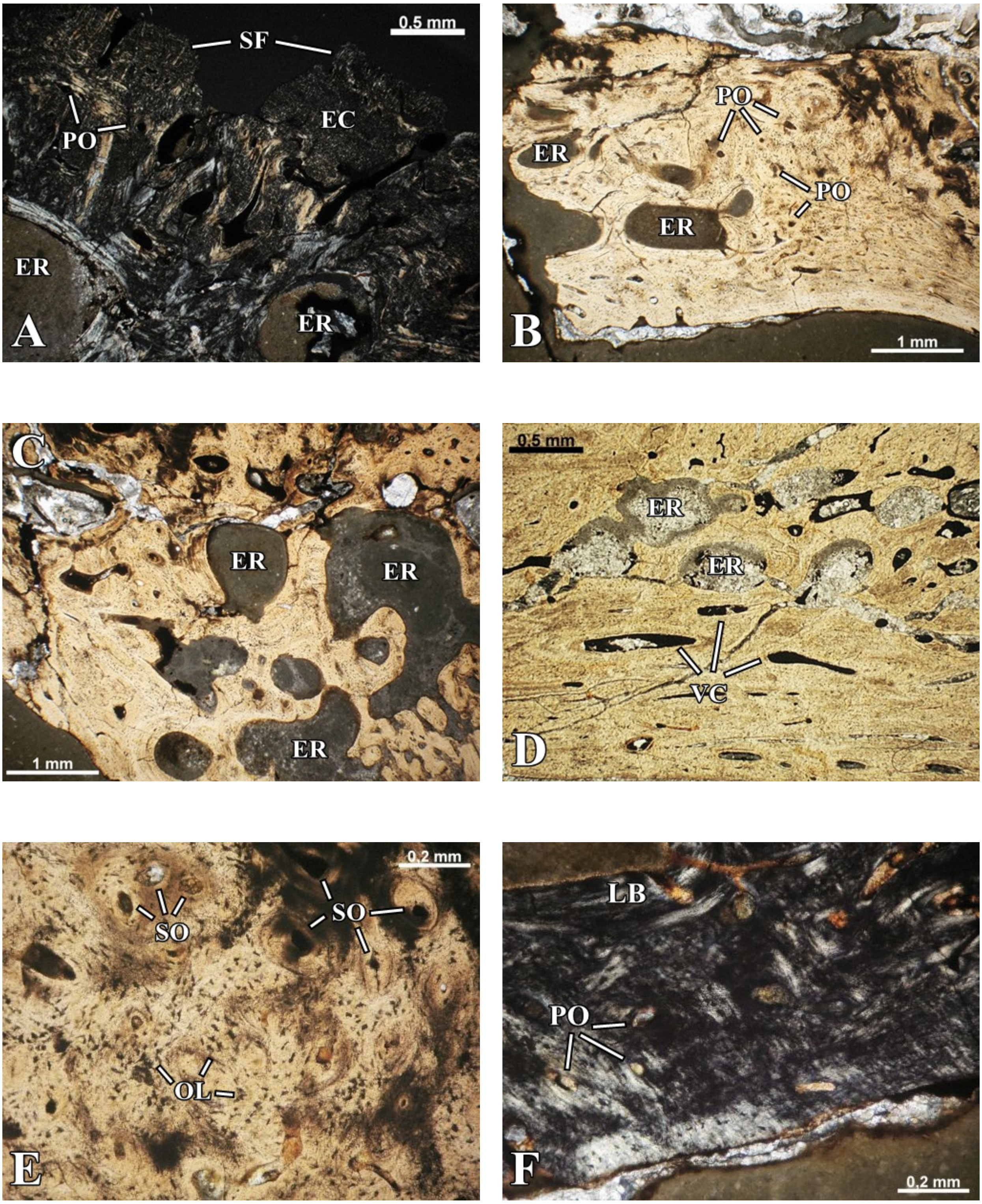

Fig. 2. The histological details of the premaxilla of *Metoposaurus krasiejowensis*. **(A)** External cortex with distinct Sharpey's fibers in the area of sculptural ridges; image in CPL; **(B-C)** Middle region with numerous and large erosion cavities; image in PPL; **(D)** Vascular canals in the upper parts of middle region; image in PPL; **(E)** The primary and secondary osteons visible in the middle region; image in PPL; **(F)** Middle region and internal cortex; image in CPL. Abbreviations: SF = Sharpey's fibers, EC = external cortex, PO = primary osteons, SO =

secondary osteons, ER = erosion cavities, VC = vascular canals, OL = osteocyte lacunae, LB = lamellar bone, PPL = plane polarized light, CPL = cross polarized light.

Maxilla

The maxilla is built up from two branches, the dorsal one, with ornamented external cortex and ventro-lateral with dental shelf. The external surface of the tested bone is destroyed and the details of the ornamentation are not preserved (Fig. 3). Bone thickness of the dorsal branch varies from 4 mm to 12 mm, and the ratio E:M:I is 1:1.1:0.9.

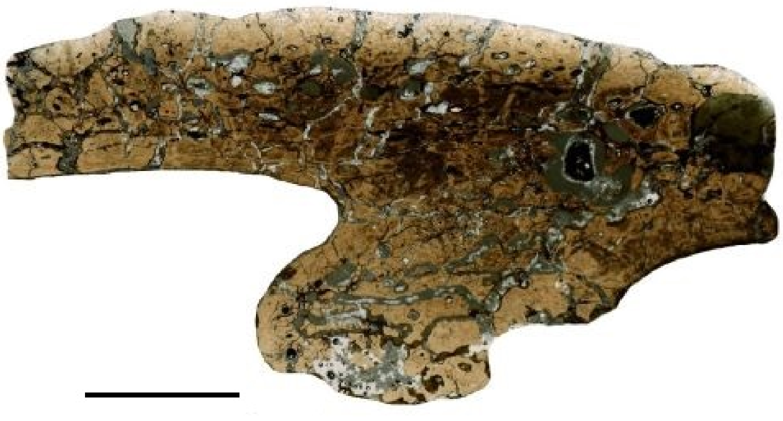

Fig. 3. Histological section of the right maxilla. Scale bar equals 5 mm.

External cortex - The remnant of the cortex consists of relatively well vascularized parallel-fibered bone (Fig. 4A). The primary osteons are less numerous than the simple vascular canals (Fig. 4B, C). The growth marks are not visible. Sharpey's fibers are relatively short but very numerous.

Middle region - The middle region is heavily cracked, however, the remains of well vascularized, cancellous bone are visible. There are numerous primary osteons, about 100 µm in diameter, and much larger secondary osteons, about 150 µm in diameter present (Fig. 4D). The simple vascular canals, various in shapes are mostly located next to the borders between the middle region with the external and internal cortexes. A significant part of the middle region is strongly eroded. The irregular erosion cavities are numerous and vary in sizes, from

about 370 µm up to over 2000 µm in diameter. Inside the erosion cavities the secondary deposited lamellar bone is present. The elliptically elongated osteocyte lacunae can be observed.

Internal cortex - The internal cortex about 440 µm thick, consists of parallel-fibered bone, is much thinner than the external cortex (Fig. 4E). The degree of vascularization is relatively moderate. In some areas, the cortex is avascular, while in other areas numerous accumulations of simple vascular canals and primary/secondary osteons can be observed. The vascular canals are mostly oriented parallel to the bone surface (Fig. 4F). They are usually about 200 µm long. The osteocyte lacunae are very numerous and slightly elongated. The Sharpey’s fibers are not present.

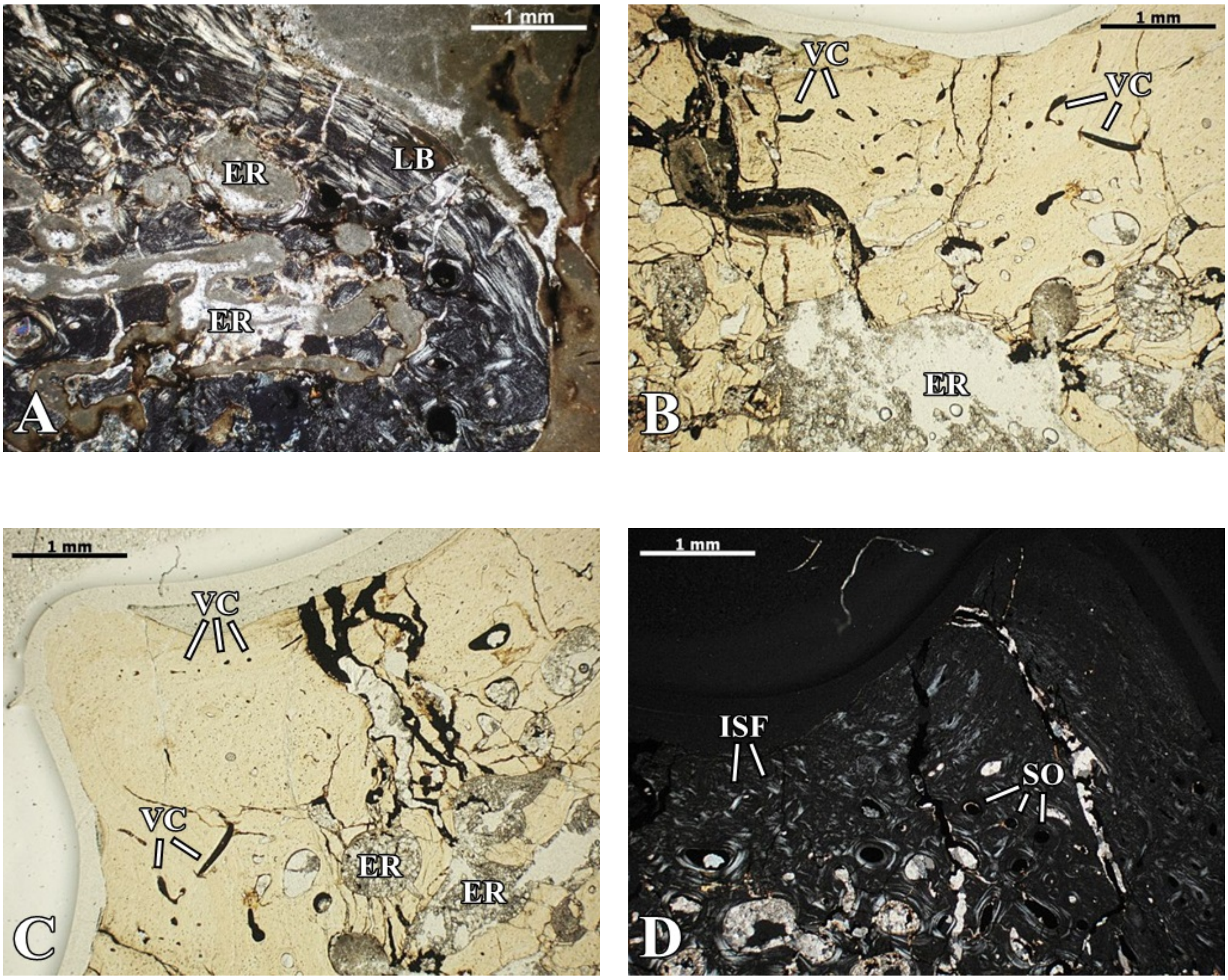

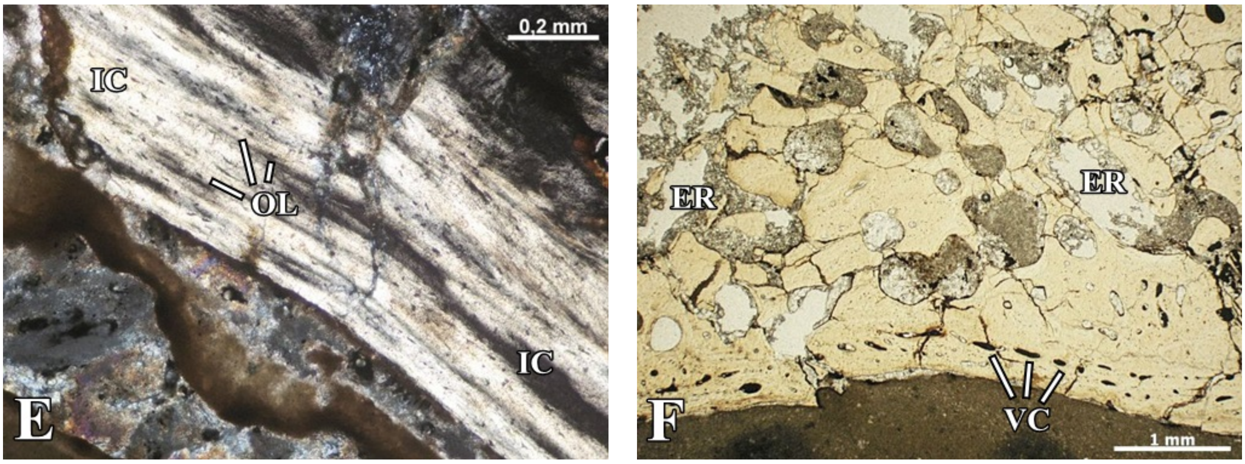

Fig. 4. The histological details of the maxilla of *Metoposaurus krasiejowensis*. **(A)** Distinct parallel-fibered bone, which create the external cortex; image in CPL; **(B-C)** Vascular canals in the area of sculptural ridges and valleys; image in PPL; **(D)** External cortex and middle region, with distinct, numerous secondary osteons; image in CPL; **(E)** Avascular internal cortex with visible, numerous cell lacunae; image in CPL; **(F)** Porous middle region and well vascularized internal cortex; image in PPL. Abbreviations: PFB = parallel-fibered bone, SO = secondary osteons, ER = erosion cavities, VC = vascular canals, ISF = Interwoven Structural Fiber bundles, IC = internal cortex, OL = osteocyte lacunae, PPL = plane polarized light, CPL = cross polarized light.

Nasal

The thin section was prepared from the frontal part of the right nasal. Its external surface possesses distinct ornamentation. In cross section, the sculptured ridges are moderate high (Fig. 5). Bone thickness ranges from 1 mm to about 8 mm, and the E:M:I ratio is 1:1.2:0.4.

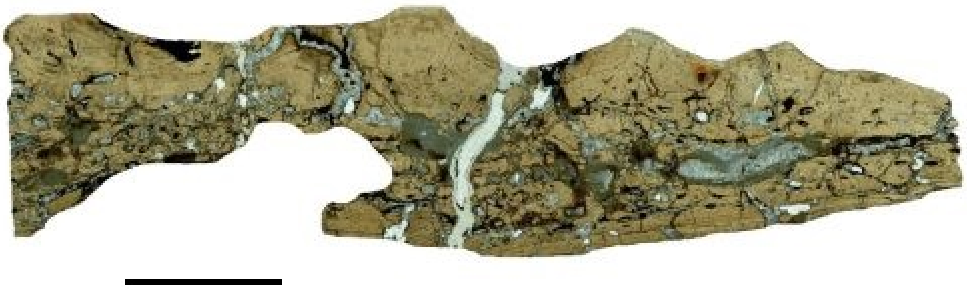

Fig. 5. Histological section of the right nasal. Scale bar equals 5 mm.

External cortex - The external cortex consists of parallel-fibered bone, with an average thickness about 270 µm. Only in the area of the ridges the cortex becomes significantly thicker, to nearly 1900 µm. The external cortex is relatively well vascularized. The simply vascular canals are present in its deeper part, and some of them may fragmentary penetrate into the middle region. Within the shallow and wide grooves, the elongated, simple vascular canals (500 µm in length) are arranged in regular rows, parallel to the bone surface. The ridges are practically avascular. Sporadically, the primary and slightly larger secondary osteons can be observed also in the deeper parts of external cortex. The numerous, short Sharpey's fibers are visible, mainly in the sculptural ridges. The rounded osteocyte lacunae are very numerous. The growth marks are not visible.

Middle region - The middle region is very vast and spongy area with well-developed vascular network (Fig. 6A). The relatively large, approximately 400 µm in length and irregularly arranged, simple vascular canals are present next the borders with external and internal cortexes. They can partially penetrate into the internal cortex. In the central part of the middle region, the simple vascular canals are almost absent, whereas very numerous secondary osteons with diameters ranging from 90 µm to 170 µm can be observed (Fig. 6B). The thickness of the lamellar bone inside the secondary osteons is 140 µm approximately. In the central part of the middle region, large and irregular erosion cavities occur, with length ranging from 300 µm to over 2500 µm (Fig. 6C). On the edges of erosion cavities, the lamellar bone of variable thickness is present. The elongate osteocyte lacunae with branching canaliculi often appear.

Internal cortex - The internal cortex consists of parallel-fibered bone, with a maximum thickness of about 490 µm. It is almost avascular, except for few simple vascular canals which partially penetrate from the middle region (Fig. 6D). The Sharpey's fibers are not visible in the internal cortex. The elliptical osteocyte lacunae (about 20 µm in length) with branched canaliculi are numerous. They typically extend in ordered rows, oriented parallel to

the collagen fibers. The growth marks are visible as an alternation of lamellar layers and two resting lines.

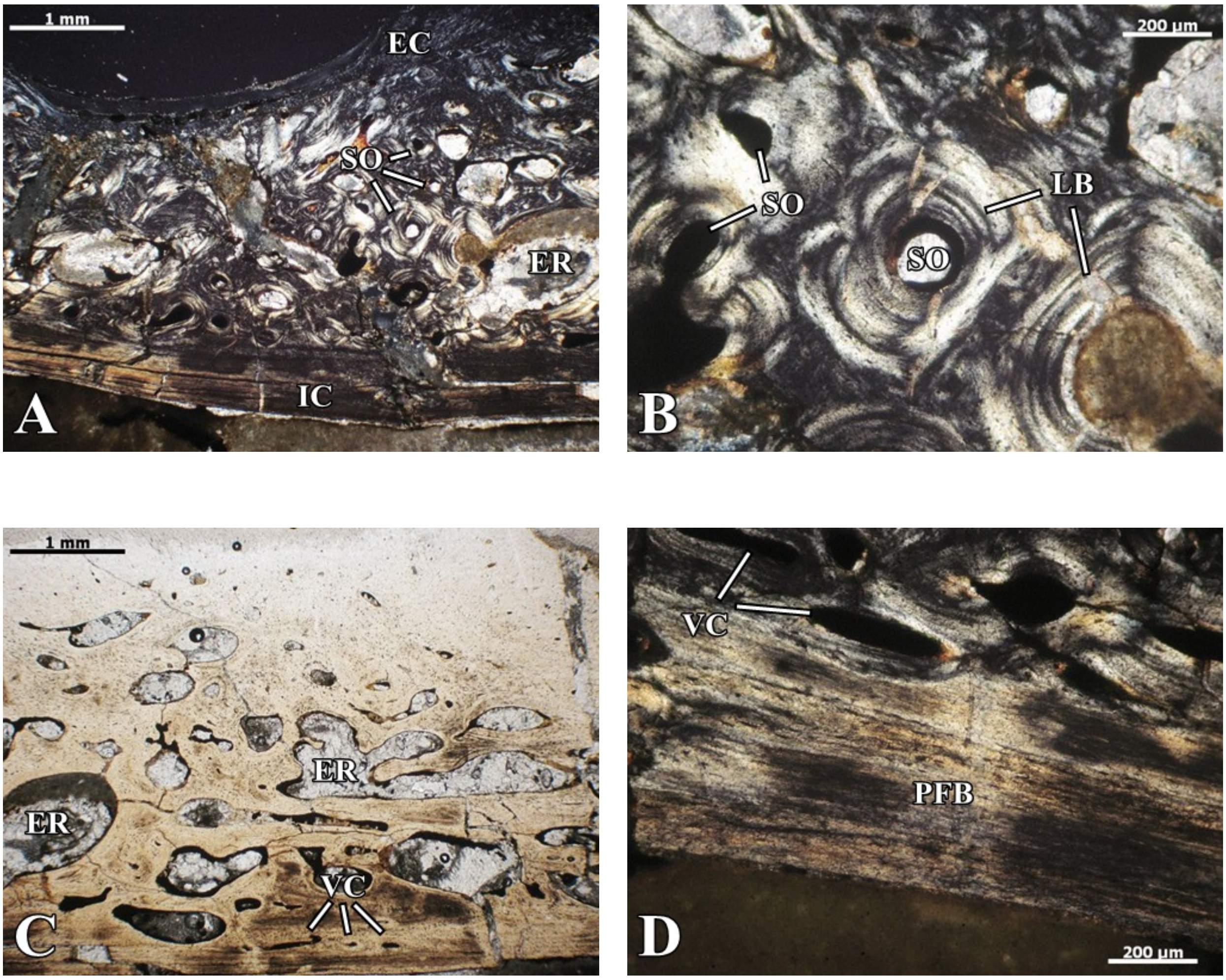

Fig. 6. The histological details of the nasal of *Metoposaurus krasiejowensis*. **(A)** Diploë structure of nasal; image in CPL; **(B)** Dense accumulation of secondary osteons in the middle region; image in CPL; **(C)** Highly eroded middle region; image in PPL; **(D)** Avascular internal cortex; image in CPL. Abbreviations: EC = external cortex, SO = secondary osteons, ER = erosion cavities, LB = lamellar bone, VC = vascular canals, IC = internal cortex, PFB = parallel-fibered bone, PPL = plane polarized light, CPL = cross polarized light.

Lacrimal

The histological thin section was prepared from the middle part of the right lacrimal, above the edge of the orbit (Fig. 7). The external surface of the lacrimal bears the irregular

sculpture of medium high (0.5 mm), steep ridges and wide grooves. The average bone thickness in cross-section is 2.7 mm, and the ratio E:M:I is 1:1.6:0.7.

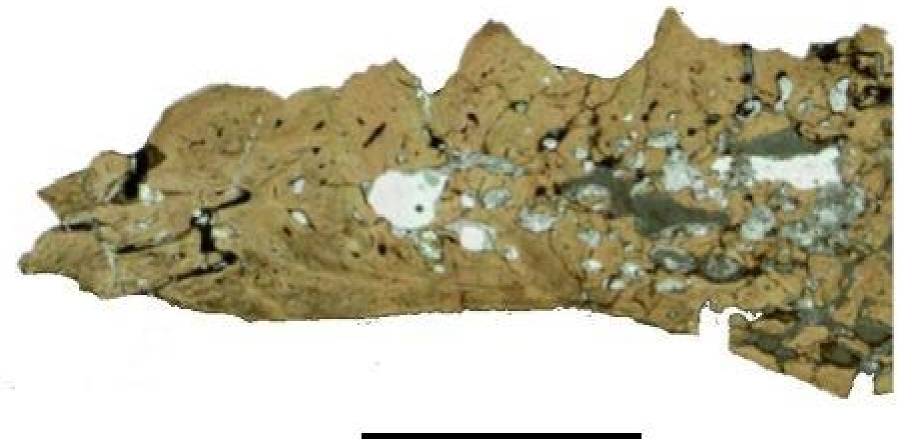

Fig. 7. Histological section of the right lacrimal. Scale bar equals 5 mm.

External cortex - The variable in thickness external cortex consists of parallel-fibered bone (Fig. 8A). The cortex is the thickest within the ridges, up to 430 µm, while in the sculptural grooves the bone thickness is approximately 130 µm. The external cortex is well vascularized with numerous, anastomosing simple vascular canals. These canals are more numerous in ridges then in grooves (Fig. 8B & 8C). The thin (about 10 µm in diameter), well mineralized Sharpey's fibers are packed in bundles. They occur within sculptural ridges and on sutural edges. In the upper parts of ridges, the distinct growth marks manifested as a sequence of thin resting lines and layers of parallel-fibered bone are present. The irregularly arranged osteocyte lacunae possess branched canaliculi.

Middle region - In its central parts large, ranging from 320 µm to almost 2500 µm in diameter, erosion cavities are present (Fig. 8D). The edges of the erosion cavities are partially surrounded by the thin layer of lamellar bone. The degree of vascularization of the middle region is relatively high, the irregularly arranged simple vascular canals and secondary osteons, about 110 µm in diameter, are very numerous (Fig. 8E). In some parts of the middle

region, the small secondary osteons occur in dense clusters (Fig. 8F). The elliptically elongated osteocyte lacunae, approximately 20 µm in length, are numerous.

Internal cortex - The internal cortex, with variable thickness, reaching up to 1000 µm, consists of parallel-fibered bone. It is relatively well vascularized. The simple vascular canals are arranged in several rows, orientated with its long axis at an angle of about 45^0 with respect to the cortex. The secondary osteons appear very rarely. Elliptical osteocyte lacunae are very numerous and irregularly arranged in the bone matrix.

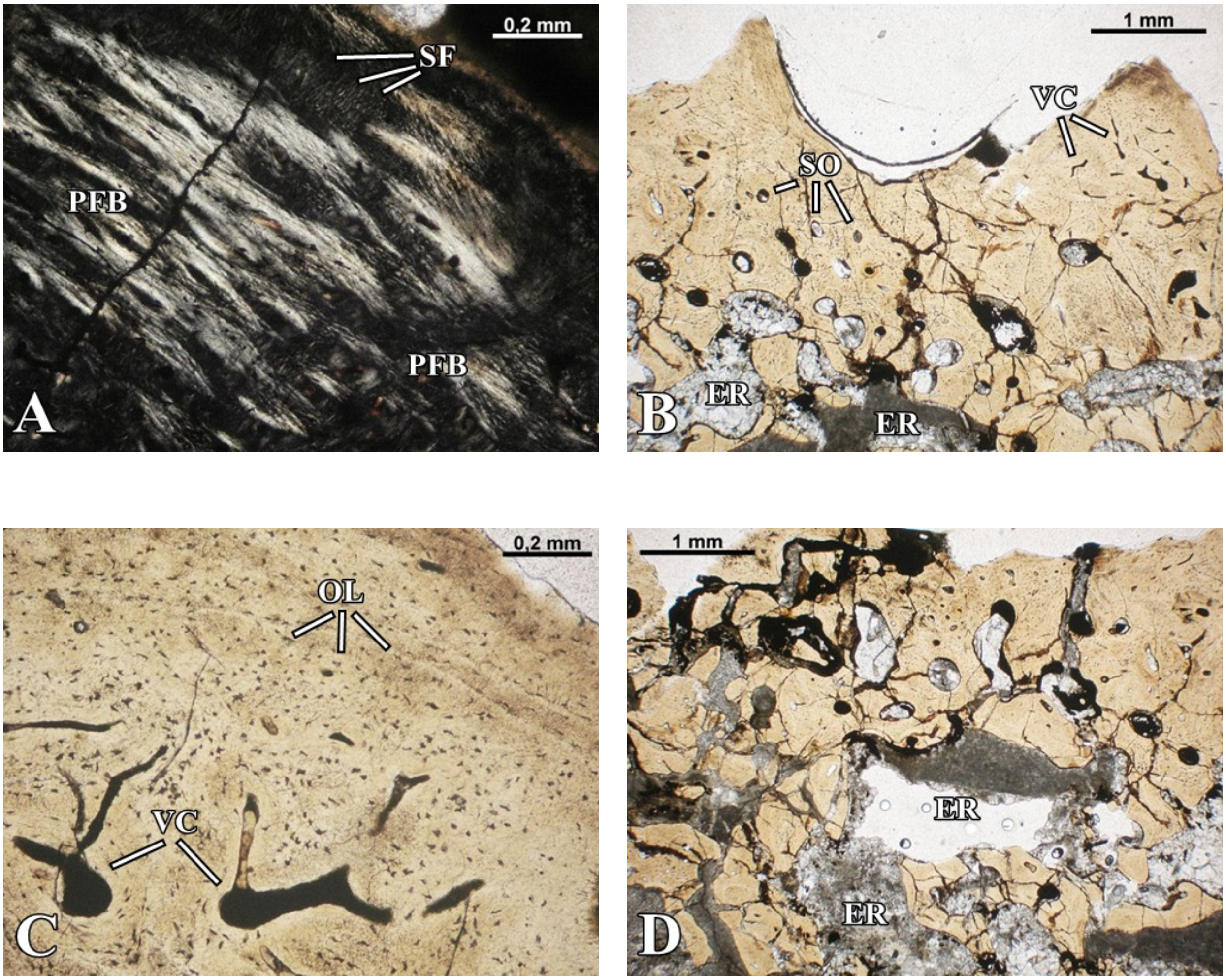

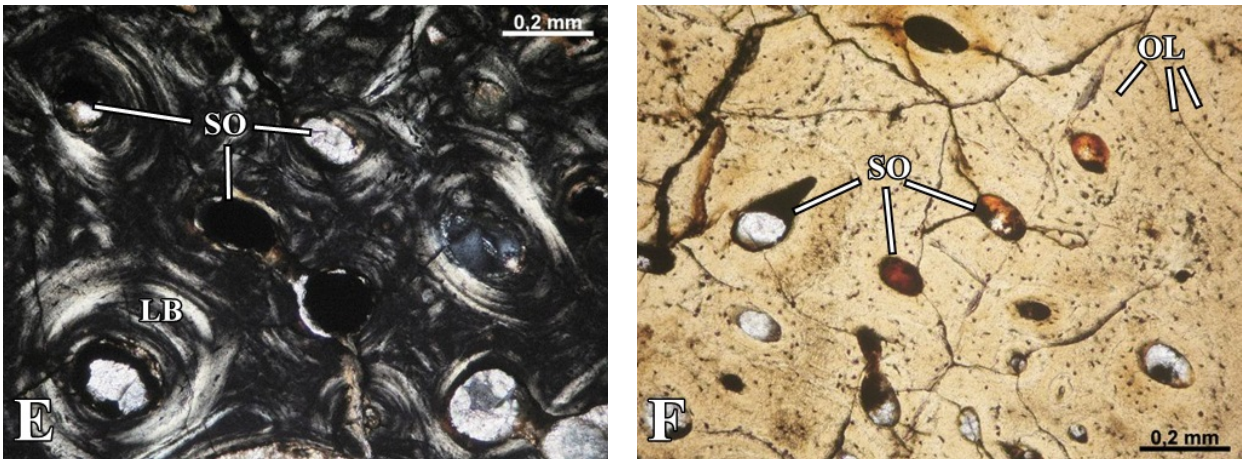

Fig. 8. The histological details of the lacrimal of *Metoposaurus krasiejowensis*. **(A)** Parallel-fibered bone and short bundles of Sharpey's fibers in external cortex; image in CPL; **(B)** Ornamented and well vascularized external surface of lacrimal; image in PPL; **(C)** Vascular canals in external cortex; image in PPL; **(D)** Middle region with large erosion cavities; image in PPL; **(E-F)** Dense accumulation of secondary osteons in the middle region; images in CPL (E) and PPL (F). Abbreviations: PFB = parallel-fibered bone, SF = Sharpey's fibers, EC = external cortex, SO = secondary osteons, ER = erosion cavities, VC = vascular canals, LB = lamellar bone, OL = osteocyte lacunae, PPL = plane polarized light, CPL = cross polarized light.

Prefrontal

The investigated section of the left prefrontal was prepared from the middle part of this bone (Fig. 9). The external surface of the bone possesses distinct ornamentation – high and steep ridges and deep grooves. Sectional bone thickness is about 5 mm, and the ratio E:M:I is 1:1.7:0.8.

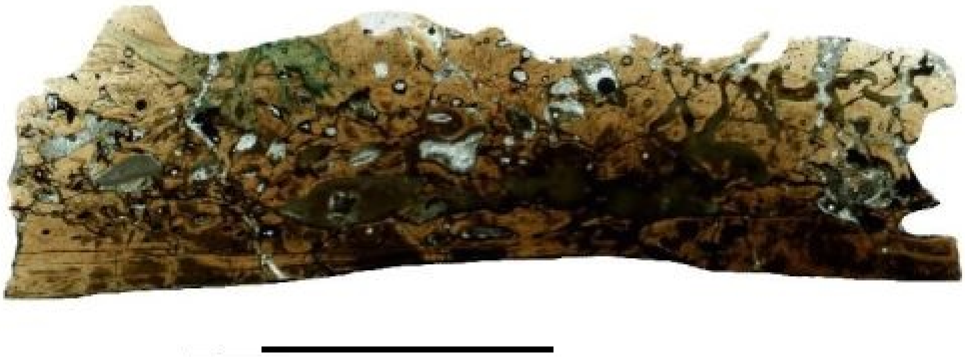

Fig. 9. Histological section of the left prefrontal. Scale bar equals 5 mm.

External cortex - The fragmentarily preserved external cortex consists of parallel-fibered bone. The irregular simple vascular canals appear within the sculptural grooves. The primary osteons are less numerous than simple vascular canals. The Sharpey's fibers occasionally appear, especially in areas where the sculptural grooves are shallower. The elongated osteocyte lacunae with branched canaliculi are very numerous in the bone matrix. The growth marks are not present.

Middle region - The vast middle region consists of partially eroded cancellous bone. The erosion cavities are various in sizes, ranging from 280 μm to about 2000 μm (Fig. 10A, B). They are relatively small in upper parts of middle region, while in the deeper part they are significantly larger. The lamellar bone partially surrounds their edges. The simple vascular canals are not numerous. The primary and secondary osteons, with a diameter about 100 μm, are more numerous (Fig. 10C). The round osteocyte lacunae are as numerous as in the external cortex.

Internal cortex - The internal cortex consists of parallel-fibered bone, with variable thickness up to 650 μm. The entire internal cortex is poorly vascularized (Fig. 10D). The simple vascular canals are not visible and the primary osteons, with diameters about 130 μm, are rarely. The Sharpey's fibers are not present. The elliptically elongated osteocyte lacunae with branched canaliculi are not as numerous as in external cortex and middle region.

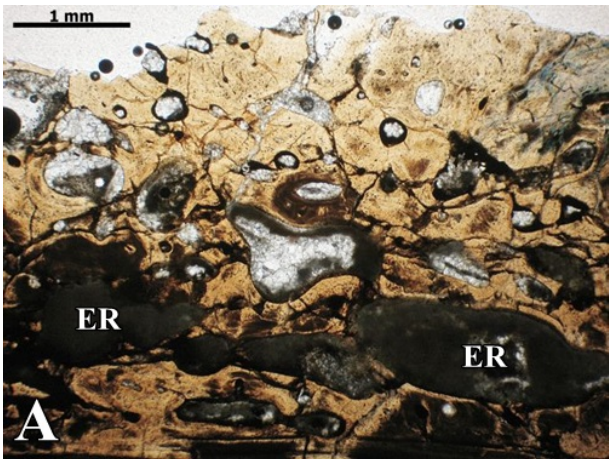

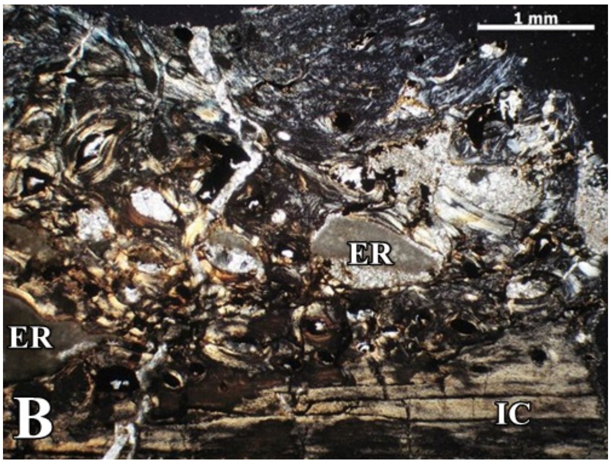

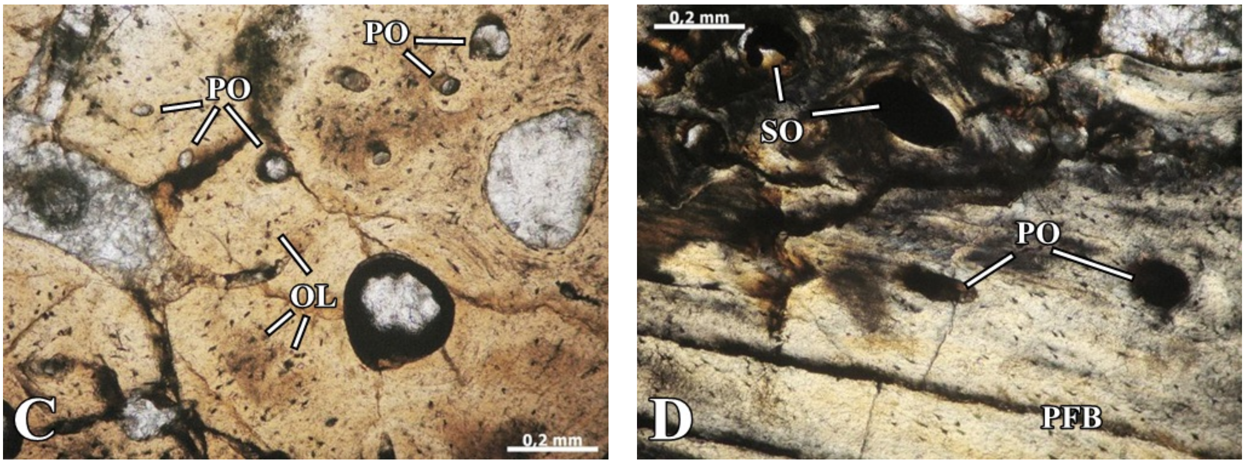

Fig. 10. The histological details of the prefrontal of *Metoposaurus krasiejowensis*. **(A-B)** Middle region with large erosion cavities; images in CPP (A) and PPL (B); **(C)** Small primary osteons and numerous osteocyte lacunae; image in PPL; **(D)** Part of internal cortex with visible secondary and primary osteons; image in CPL. Abbreviations: ER = erosion cavities, PO = primary osteons; SO = secondary osteons, PFB = parallel-fibered bone, IC = internal cortex, OL = osteocyte lacunae, PPL = plane polarized light, CPL = cross polarized light.

Jugal & Postorbital

The histological sample was made from the left part of the skull. The external surface of jugal and postorbital is sculptured by high (of about 1 mm) ridges and deep, wide grooves (Fig. 11). The average thickness of the sectioned bones is 5.9 mm and the ratio E:M:I is 1:1.5:0.7.

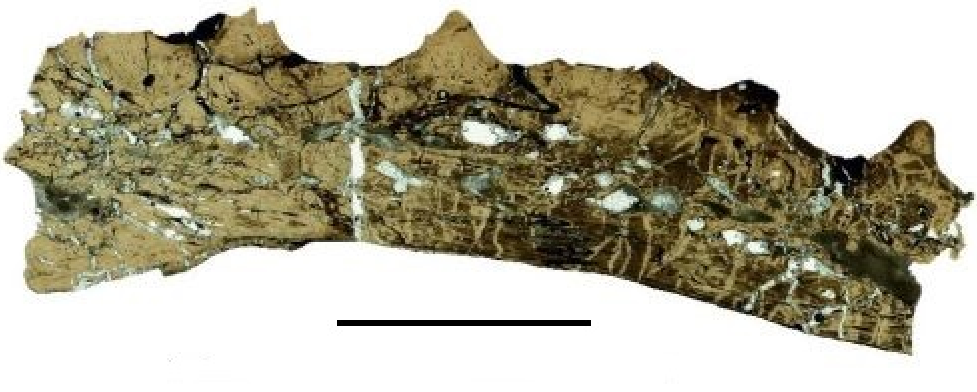

Fig. 11. Histological section of the left jugal and the postorbital. Scale bar equals 5 mm.

External cortex - The well vascularized external cortex with relatively high thickness consists of parallel-fibered bone. The very numerous, irregularly arranged simple vascular canals are grouped in some fragments of cortex. In the central parts of the ridges, the simple vascular canals are elongated and relatively small (about 80 µm), while deeper they are almost double in length (Fig. 12A, D). The round simple vascular canals are arranged in several regular rows in the ridges (Fig. 12B). In the outermost row the simple canals are very small, about 60 µm in diameter, while deeper they become more elongated to about 150 µm in length. The not numerous small primary osteons occur mainly in the deeper parts of the external cortex. A short, well mineralized and packed in bundles Sharpey's fibers, with diameter about 10 µm, are observed next to the surface and on the sutural edges (Fig. 12C). Within the sculptural ridges of jugal, the growth marks, manifested as a sequence of thin resting lines and the layers of parallel-fibered bone, can be observed (Fig. 12E, F). In some places the alternation of valleys and ridges is preserved. The rounded and elongated osteocyte lacunae with branched canaliculi are very numerous in the entire external cortex.

Middle region - The extensive, highly eroded and well vascularized middle region consists of cancellous bone. The elongated, simple vascular canals are less numerous than in the external cortex. The large secondary osteons of about 150 µm in diameter are common. The large erosion cavities, with lengths up to 1800 µm occur in the deeper parts of the middle region (Fig. 12G). The thickness of irregularly arranged trabeculae varies from 160 µm to over 450 µm. The interiors of the erosion cavities are partially lined by discontinuous layer of the lamellar bone. The middle region is heavily cracked (Fig. 12H). The slightly elongated osteocyte lacunae are very numerous.

Internal cortex - The internal cortex with a relatively constant thickness consists of parallel-fibered bone. Only in jugal, the cortex becomes slightly thicker. The vascular network is poorly developed, but in some places the simple vascular canals can be observed. The long axes of the numerous and extending radially simple vascular canals (100 µm in length) are

orientated an angle of about 80^0 relative to the middle region (Fig. 12I). The secondary osteons are not numerous. The Sharpey’s fibers are not visible. The elongated osteocyte lacunae are very numerous. They are oriented parallel to the collagen fibers and possess branched canaliculi (Fig. 12J). The growth marks are visible in jugal as an alternation of lamellar layers and three resting lines.

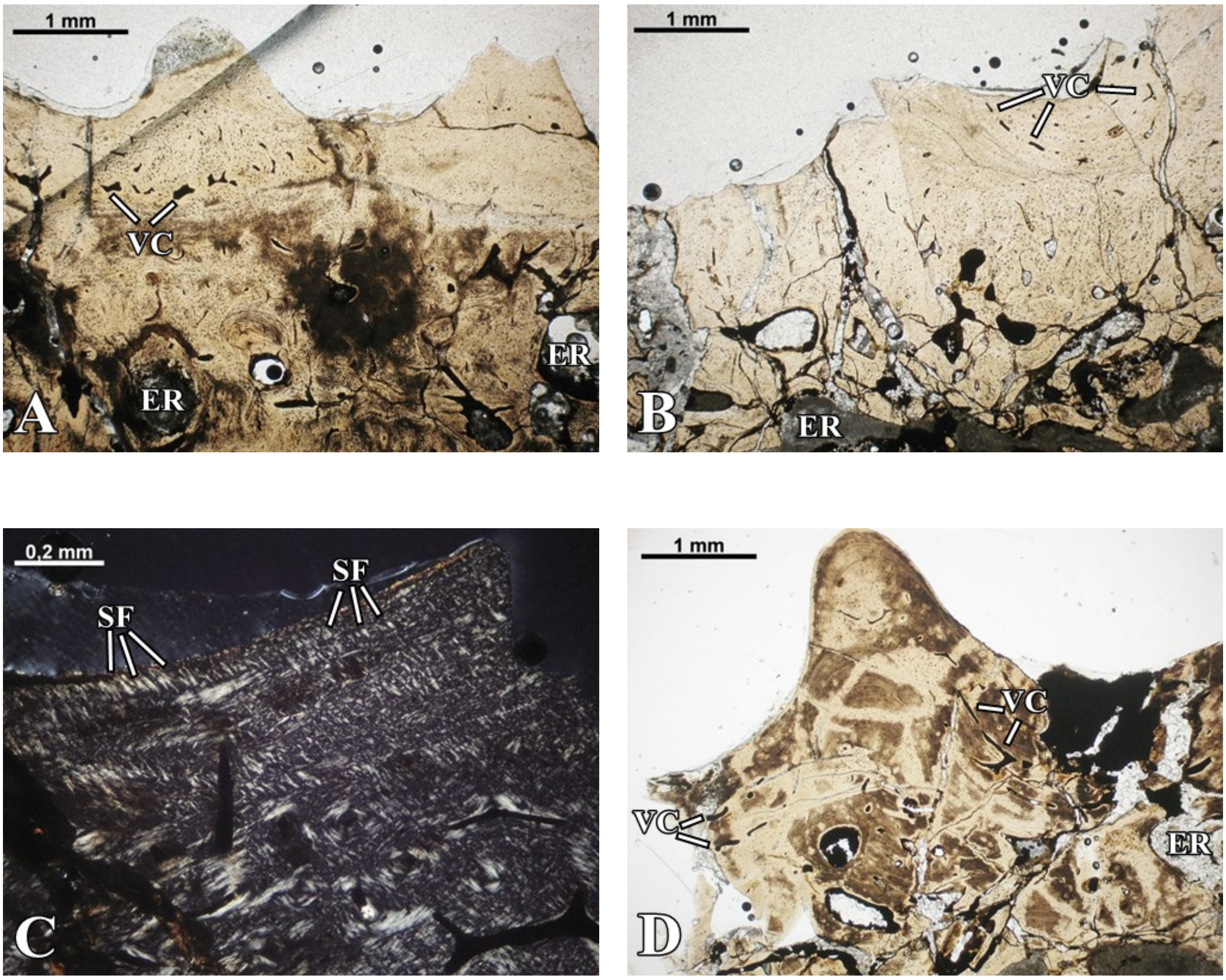

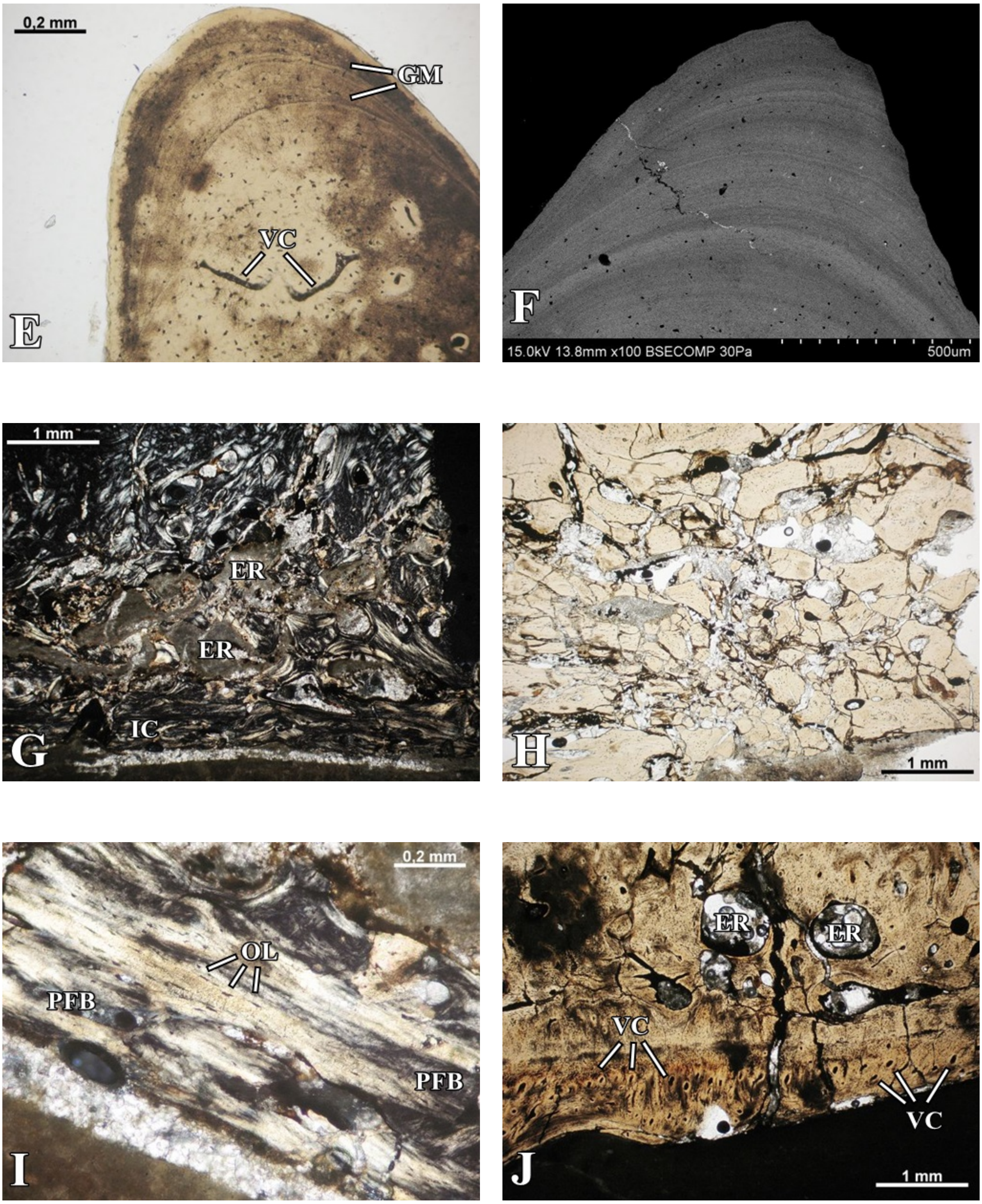

Fig. 12. The histological details of the jugal and the postorbital of *Metoposaurus krasiejowensis*. **(A-B, D)** Well vascularized external surface in the jugal and the postorbital; image in PPL; **(C)** Dense bundles of well mineralized Sharpey's fibers in the external cortex; image in CPL; **(E-F)** Numerous growth marks structure in the area of sculptural ridges; images in PPL (E) and in scanning electron microscope (F); **(G-H)** Heavily eroded and cracked middle region; image in CPL (G) and PPL (H); **(I)** Parallel-fibered bone with distinct, numerous

osteocyte lacunae; image in CPL; **(J)** Well vascularized part of internal cortex; image in PPL. Abbreviations: VC = vascular canals, ER = erosion cavities, SF = Sharpey's fibers, GM = growth marks structure, PFB = parallel-fibered bone, OL = osteocyte lacunae, PPL = plane polarized light, CPL = cross polarized light.

Postfrontal

The histological sample was prepared from the left postfrontal, behind the posterior edge of the orbit (Fig. 13). The clear ornamentation of external surface consists of about 0.3 mm high ridges and shallow grooves. The bone thickness ranges from 3 mm to 5 mm and the ratio E:M:I is 1:1.7:0.6.

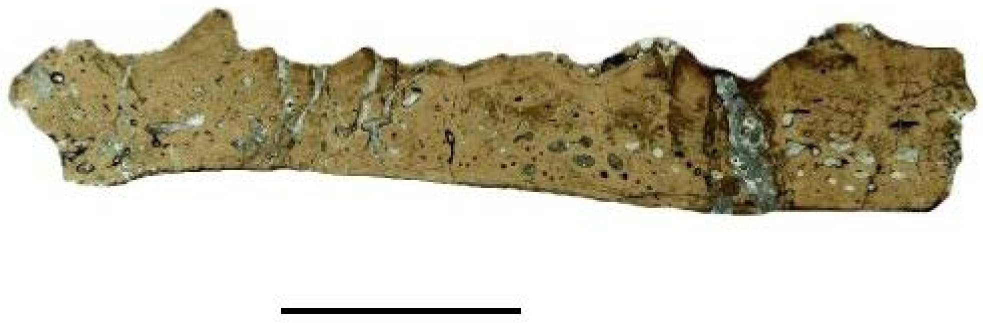

Fig. 13. Histological section of the left postfrontal. Scale bar equals 5 mm.

External cortex - The well vascularized external cortex, with a thickness of about 440 μm, consists of parallel-fibered bone. The irregularly arranged simple vascular canals are very numerous. Within the sculptural grooves, the simple vascular canals are strongly elongated to even 400 μm in length. The small and rare primary osteons occur especially in the deeper parts of the external cortex. The preserved fragments of the sculptural ridges possess the cyclical growth marks, manifested as a sequence of thin resting lines and layers of parallel-fibered bone, which follow the shape of the ridges. In some areas the alternation of valleys and ridges is preserved. The rounded osteocyte lacunae are very numerous in the entire cortex. The Sharpey's fibers are rare. The distinct collagen fibers create a thick Interwoven Structural Fibers (IFS).

Middle region - The middle region consists of cancellous bone, but the erosion cavities are small, up to 450 µm, and appear sporadically (Fig. 14A, B). Their irregular edges are partially lined by layers of lamellar bone. The elongated simple vascular canals can be observed next to the border with the external cortex. The small primary osteons, with a diameter of about 70 µm, and larger, about 120 µm secondary osteons are very numerous (Fig. 14C). The thickness of the layer of lamellar bone inside secondary osteons is 70 µm. In some parts of the bone, the small secondary osteons form Haversian tissue. The elongated osteocyte lacunae with diameter about 10 µm are randomly arranged in the bone matrix.

Internal cortex - The thin, almost avascular internal cortex consists of parallel-fibered bone (Fig. 14D). Its average thickness is 240 µm. The osteocyte lacunae are less numerous then in the external and middle regions. They are elongated and directed parallel to the long axis of the internal cortex. The Sharpey's fibers are not present. The growth marks are visible as the alternation of lamellar layers and four resting lines.

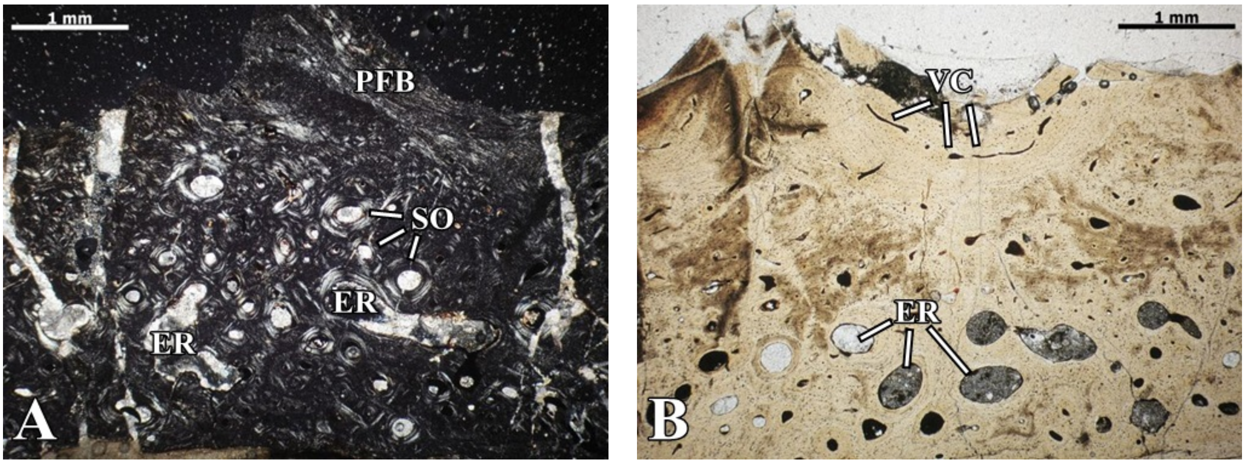

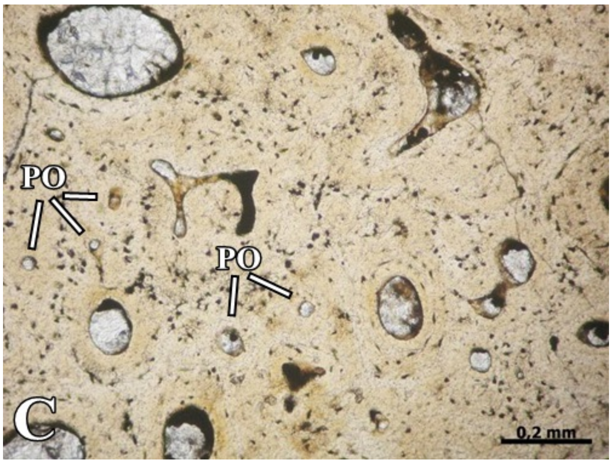

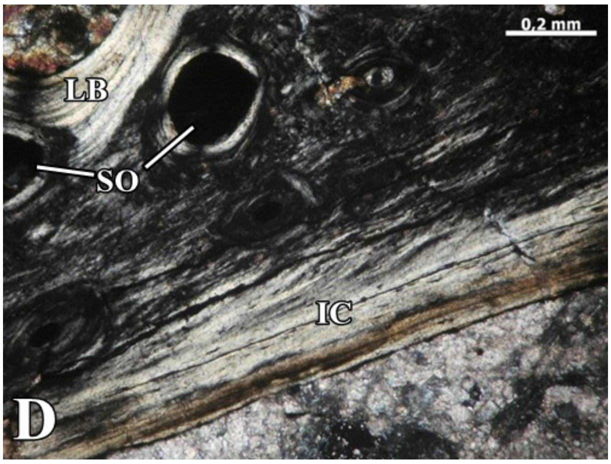

Fig. 14. The histological details of the postfrontal of *Metoposaurus krasiejowensis*. **(A-B)** External cortex and middle region in CPL (A) and PPL (B); **(C)** Small and numerous primary osteons in middle region; image in PPL; **(D)** Thin, avascular internal cortex; image in CPL. Abbreviations: PFB = parallel-fibered bone, SO = secondary osteons, PO = primary osteons, ER = erosion cavities, VC = vascular canals, LB = lamellar bone, IC = internal cortex, PPL = plane polarized light, CPL = cross polarized light.

Frontal

The histological sample was prepared from the middle part of the right frontal (Fig. 15). The dermal sculpture of frontal is composed of high ridges and wide grooves. The average bone thickness is about 4.7 mm, and the ratio E:M:I is 1:1.5:0.6.

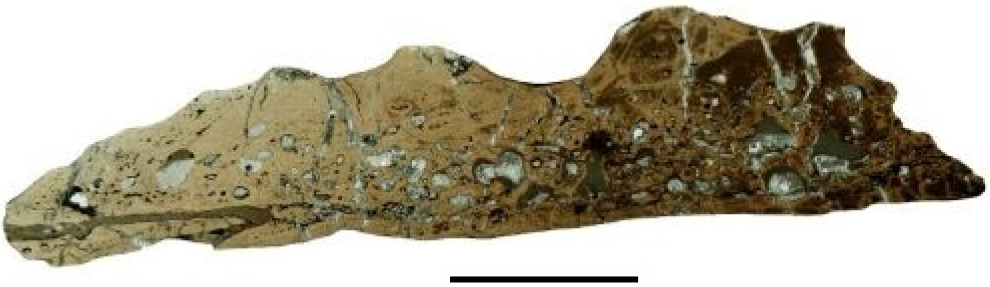

Fig. 15. Histological section of the right frontal. Scale bar equals 5 mm.

External cortex - The external cortex consists of parallel-fibered bone with a thickness approximately 660 µm. The cortex is relatively poor vascularized (Fig. 16A). The

small and irregular simple vascular canals are present in deeper areas of external cortex, next to the border with the middle region. The primary and secondary osteons are not visible. The Sharpey's fibers are short and rare. They appear occasionally in the cortex only as very short bundles and are more numerous on the edges of cranial suture. The growth marks, manifested as a sequence of thin resting lines and the layers of parallel-fibered bone, occur in the apexes of the sculptural ridges and in the ridges. They follow the shape of the ridges and grooves (Fig. 16B). The rounded osteocyte lacunae of about 10 µm in diameter and with branched canaliculi are numerous in the entire cortex.

Middle region - Well vascularized and highly eroded middle region consists of cancellous bone. The irregular in shape and different in size simple vascular canals are very numerous. In upper part of middle region the amount of simple vascular canals increases. The numerous secondary osteons, about 180 µm in diameter, do not form a Haversian tissue. The erosion cavities, from 360 µm to over 1350 µm in length, are present in the large amount in the middle region (Fig. 16C). The rounded bone cell lacunae with preserved canaliculi are more numerous in the middle region than in the external cortex.

Internal cortex - The thin internal cortex consists of parallel-fibered bone (Fig. 16D). The average thickness of cortex is 420 µm. The vascularization of the internal cortex is moderate. The longitudinal, simple vascular canals appear more frequently than the secondary osteons. The Sharpey's fibers cannot be observed. The elliptically elongated osteocyte lacunae with a diameter about 20 µm are as numerous as in external cortex. They are oriented in regular rows parallel to the cortex fibers.

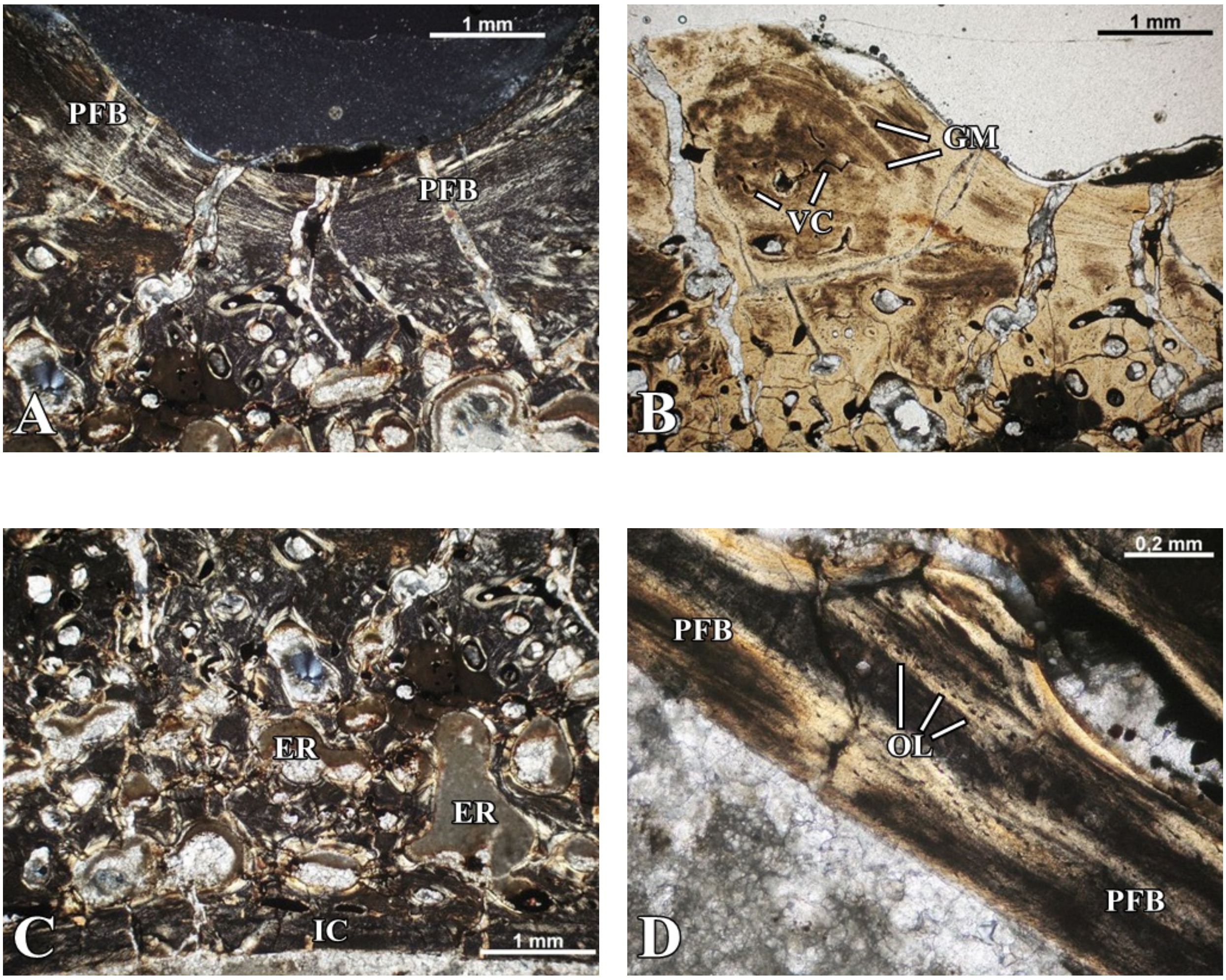

Fig. 16. The histological details of the frontal of *Metoposaurus krasiejowensis*. **(A)** Thick layer of parallel-fibered bone within the sculptural ridges; image in CPL; **(B)** Well vascularized, sculptural ridges with distinct growth marks structures; image in PPL; **(C)** Highly porous and remodeled middle region; image in CPL; **(D)** Avascular internal cortex with numerous osteocyte lacunae; image in CPL. Abbreviations: PFB = parallel-fibered bone, VC = vascular canals, GM = growth marks structure, ER = erosion cavities, IC = internal cortex, OL = osteocyte lacunae, PPL = plane polarized light, CPL = cross polarized light.

Parietal

The investigated section of the right parietal was prepared from the middle part of this bone, approximately 1.5 cm above the pineal foramen. The external surface of the parietal bears a polygonal sculpture builds from ridges about 1 mm high, and narrow pits (Fig. 17). The thickness of the bone is about 5 mm, and the ratio E:M:I is 1:1.6:0.6.

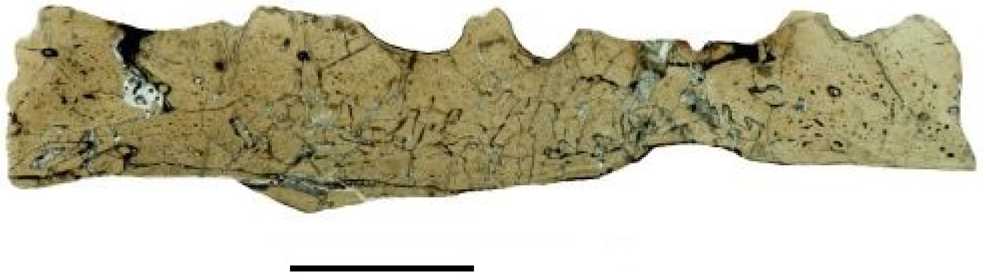

Fig. 17. Histological section of the right parietal. Scale bar equals 5 mm.

External cortex - The external cortex consists of variable in thickness parallel-fibered bone (Fig. 18A). In the grooves, the average thickness of cortex is 240 µm, while in the sculptural ridges are much thicker to over 650 µm. The simple vascular canals are very numerous, mostly in the deeper parts of the external cortex. They are strongly elongate to about 180 µm (Fig. 18B & 18C). The primary osteons are small, with diameters of about 30 µm, and they appear in much lower amounts. The few Sharpey's fibers occur occasionally in the sculptural ridges in the form of short bundles. The rounded osteocyte lacunae, with diameters of 10 µm, are numerous within the entire bone matrix. The growth marks are not visible.

Middle region - The well vascularized middle region create a vast area consists of cancellous bone. The erosion cavities are small, about 270 µm in diameter (Fig. 18D). The simple vascular canals, irregular in shape and variable in sizes, primary and secondary osteons are numerous (Fig. 18E). The secondary osteon are large, about 90 µm in diameter. The rounded and elongated osteocyte lacunae, with a diameter about 10 µm, occur in similar quantities as in the external cortex. The branched canaliculi cannot be observed.

Internal cortex - The thin internal cortex consists of parallel-fibered bone. Its thickness is usually less than 150 µm, but in some areas may be up to 250 µm. The internal cortex is avascular in the entire width (Fig. 18F). The Sharpey's fibers are not visible. The

number of the elongated osteocyte lacunae (about 20 μm in length) is considerably lower. The growth marks are visible as an alternation of lamellar layers and three resting lines.

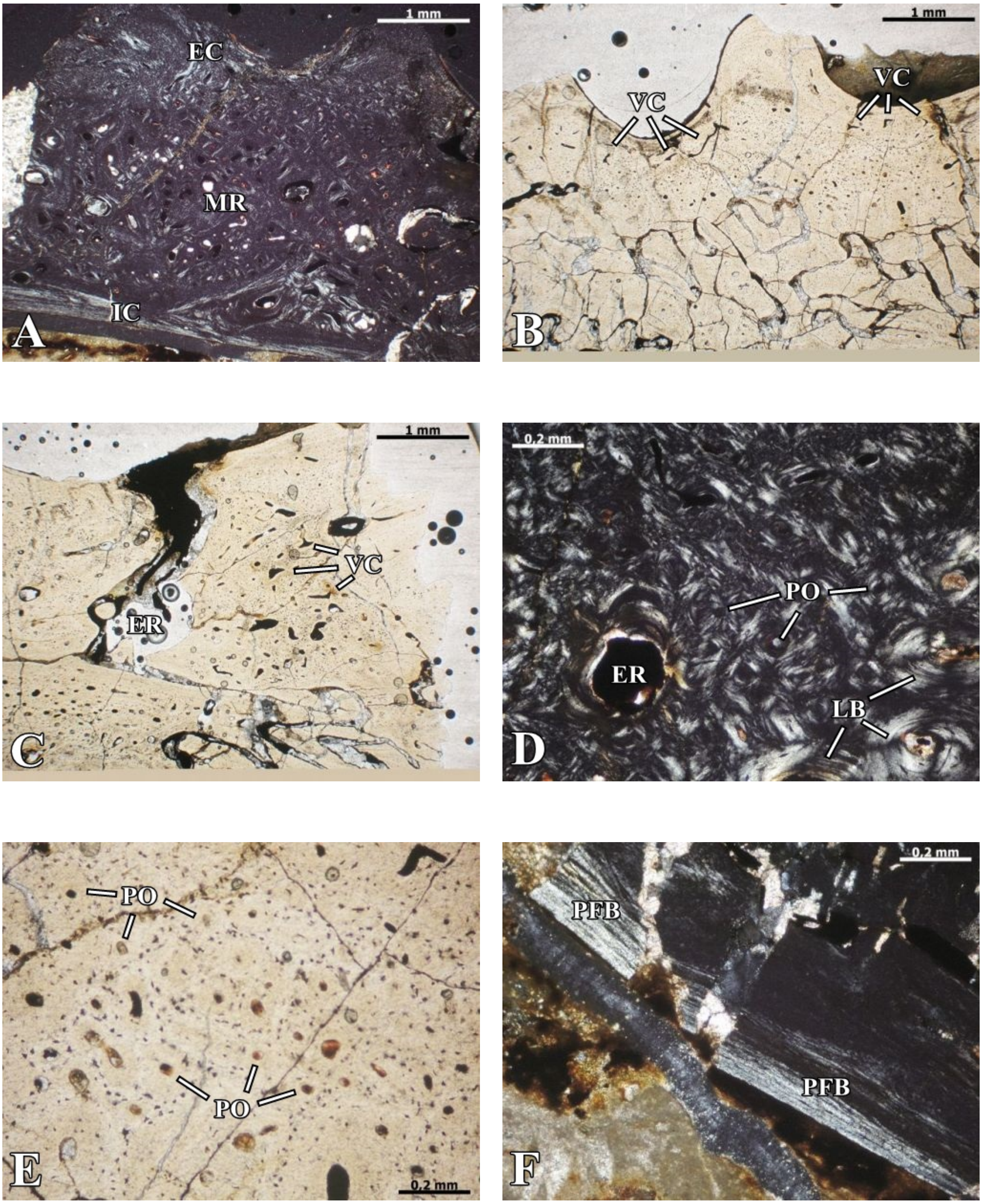

Fig. 18. The histological details of the parietal of *Metoposaurus krasiejowensis*. **(A)** Cross section of parietal, with distinct external cortex, middle region and internal cortex; image in CPL; **(B-C)** Well develop vascular network in the upper and deeper parts of external cortex; images in PPL; **(D-E)** Dense accumulation of small primary osteons in the middle region; images in CPL (D) and PPL (E); **(F)** Thin and avascular internal cortex; image in CPL. Abbreviations: EC = external cortex, MR = middle region, IC = internal cortex, VC = vascular canals, ER = erosion cavities, PO = primary osteons, LB = lamellar bone, PFB = parallel-fibered bone, PPL = plane polarized light, CPL = cross polarized light.

Supratemporal

The thin section was prepared from the anterior part of the right supratemporal (Fig. 19). The external surface of this dermal bone possesses the polygonal sculpture with high, about 1.5 mm ridges and deep grooves. The sectional bone thickness is from 3 mm to 5 mm, and the ratio E:M:I is 1:1.6:0.6.

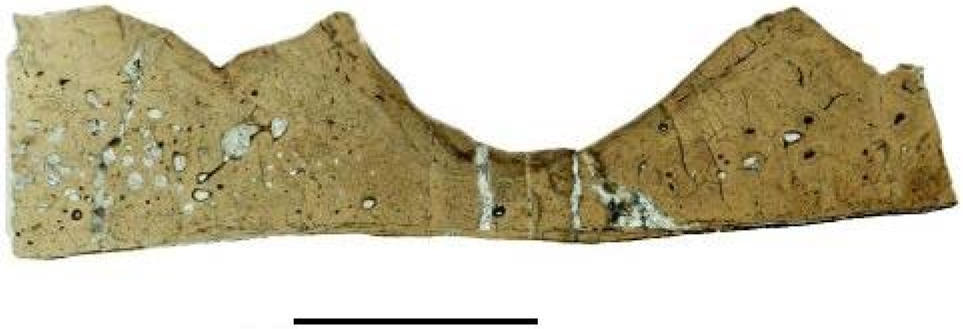

Fig. 19. Histological section of the right supratemporal. Scale bar equals 5 mm.

External cortex - The external cortex with a thickness approximately 900 µm, consists of parallel-fibered bone. The simple vascular canals, varying in size, from 50 µm to over 400 µm in length, form a dense vascular network (Fig. 20A). They are present in larger amounts within the sculptural ridges, while in the grooves they are much smaller and arranged in orderly rows (Fig. 20B & 20C). The small primary osteons appears very rarely. The short Sharpey's fibers are visible mainly in the deeper parts of the external cortex. The rounded

osteocyte lacunae with branched canaliculi are numerous. In some places the alternation of valleys and ridges is preserved.

Middle region - The external cortex transits to cancellous and well vascularized middle region. The erosion cavities, about 340 µm in diameter, are dense concentrated in some areas of the central parts of the middle region, whereas in other areas their amount is limited. The simple vascular canals and secondary osteons, ranging from 50 µm to 250 µm in diameter, are numerous (Fig. 20D). The small secondary osteons occur in densely packed clusters in some places forming the Haversian tissue (Fig. 20E). The elongated osteocyte lacunae are numerous and possess branching canaliculi.

Internal cortex - The thin, about 200 µm, internal cortex consists of parallel-fibered bone. The internal cortex is practically avascular on the entire length (Fig. 20F). The secondary osteons are small and appear very rare. The Sharpey's fibers are not visible. The elongate osteocyte lacunae of about 20 µm in length are not numerous. Their longer axes are oriented parallel to the cortex fibers. The growth marks are visible as an alternation of lamellar layers and three resting lines.

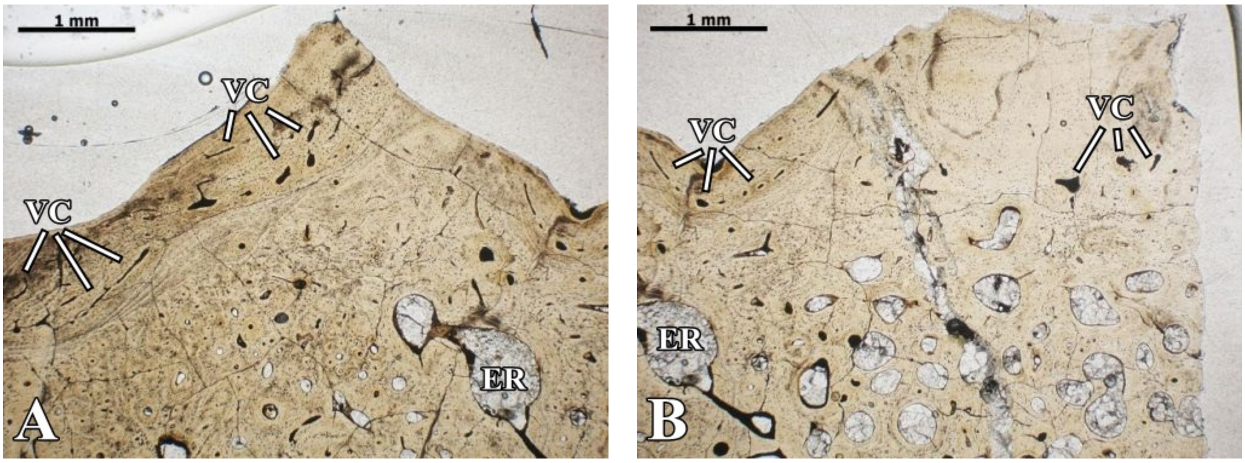

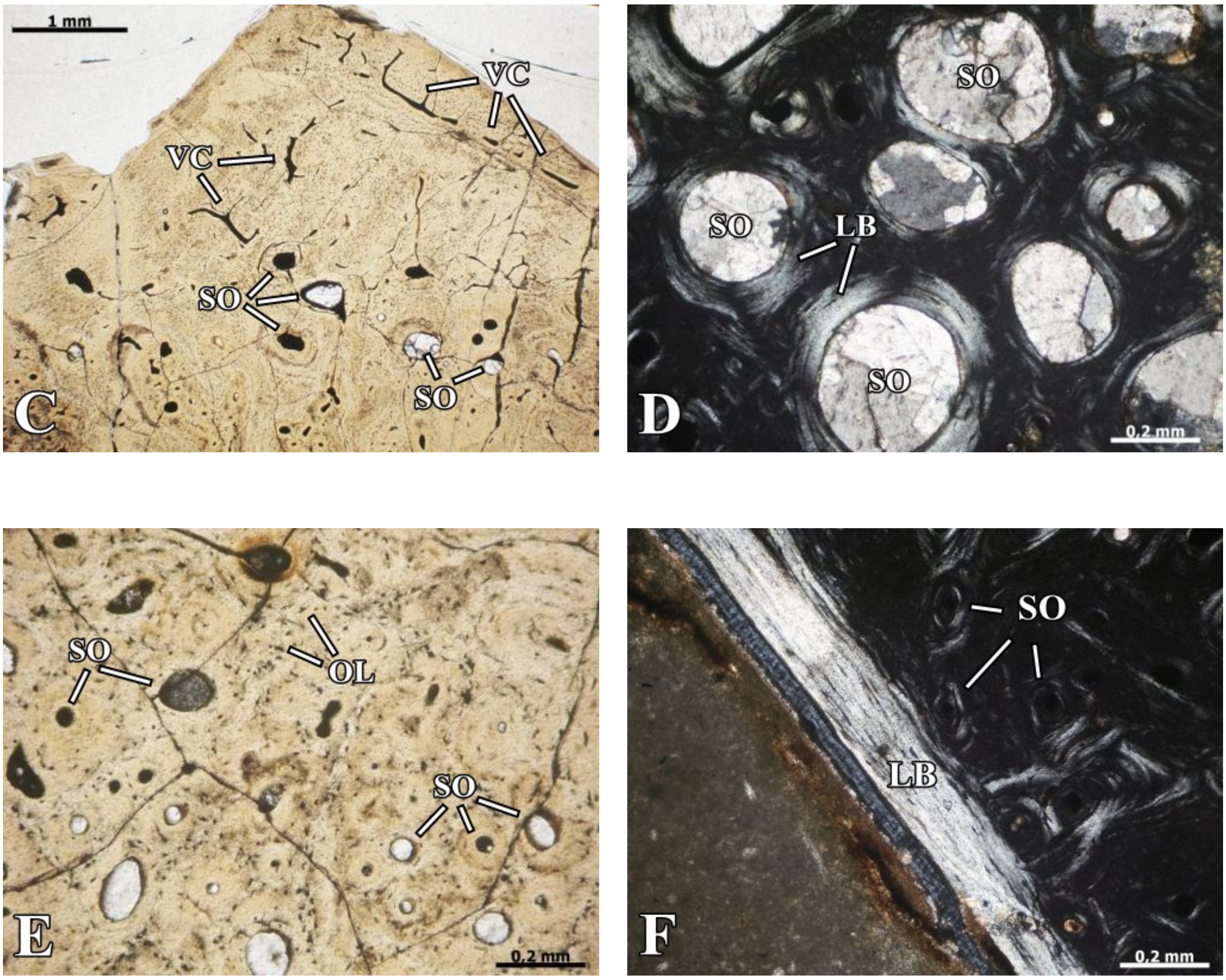

Fig. 20. The histological details of the supratemporal of *Metoposaurus krasiejowensis*. **(A-C)** Well developed vascular network in the external cortex (vascular canals) and in the middle region (secondary osteons); images in PPL; **(D)** Large and numerous secondary osteons; image in CPL; **(E)** Beginning of Haversian tissue visible in the middle region; image in PPL; **(F)** Thin and avascular internal cortex; image in CPL. Abbreviations: VC = vascular canals, ER = erosion cavities, SO = secondary osteons, LB = lamellar bone, OL = osteocyte lacunae, PPL = plane polarized light, CPL = cross polarized light.

Squamosal 1

The histological thin section was prepared from the front part of the right squamosal (Fig. 21). The external surface of the squamosal is characterized by a clear developed sculpture, consists of the high for 2 mm ridges and of the wide grooves. The bone thickness is about 5 mm, and the ratio E:M:I is 1:1.2:0.7.

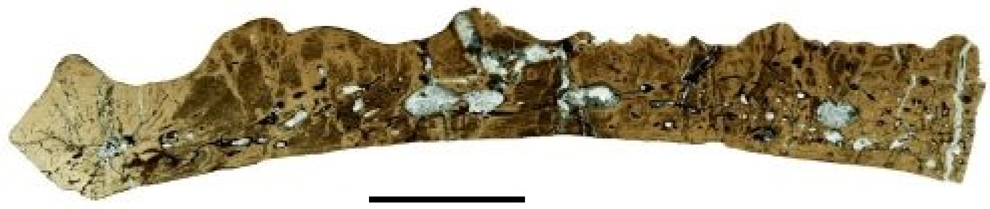

Fig. 21. Histological section of the right squamosal 1. Scale bar equals 5 mm.

External cortex - The external cortex with a maximum thickness of about 1100 µm, consists of parallel-fibered bone. The external cortex is well vascularized, mainly by the simple vascular canals irregular in shape and of variable size. The small secondary osteons, with a diameter about 30 µm, occur sporadically, mainly in the central parts of the external cortex. The thin Sharpey's fibers occur in the deeper parts of the sculptural ridges (Fig. 22A). The rounded osteocyte lacunae with branched canaliculi appear in the moderate numbers. In some places the alternation of valleys and ridges is preserved.

Middle region - The middle region is created by the well vascularized bone. The huge erosion cavities, in excess of 900 µm in diameter, are partially surrounded by the layers of the lamellar bone (Fig. 22B, C). The very numerous simple vascular canals are irregularly arranged in the bone matrix (Fig. 22D). The most numerous are, variable in size, secondary osteons. The largest, reaching up to 200 µm in diameter, are located in the central part of the middle region (Fig. 22E). In some areas, next the border with the internal cortex, the arrangement of the osteons is more orderly. The rounded osteocyte lacunae with branched canaliculi are very numerous.

Internal cortex - The internal cortex consists of parallel-fibered bone and it is much thinner than the external cortex. Its average thickness is 250 µm. The internal cortex is avascular on the entire width (Fig. 22F). The Sharpey's fibers are not visible. The osteocyte

lacunae occur sporadically. Their shape is elliptically elongated to about 20 μm, and they are arranged in the several, cyclic rows.

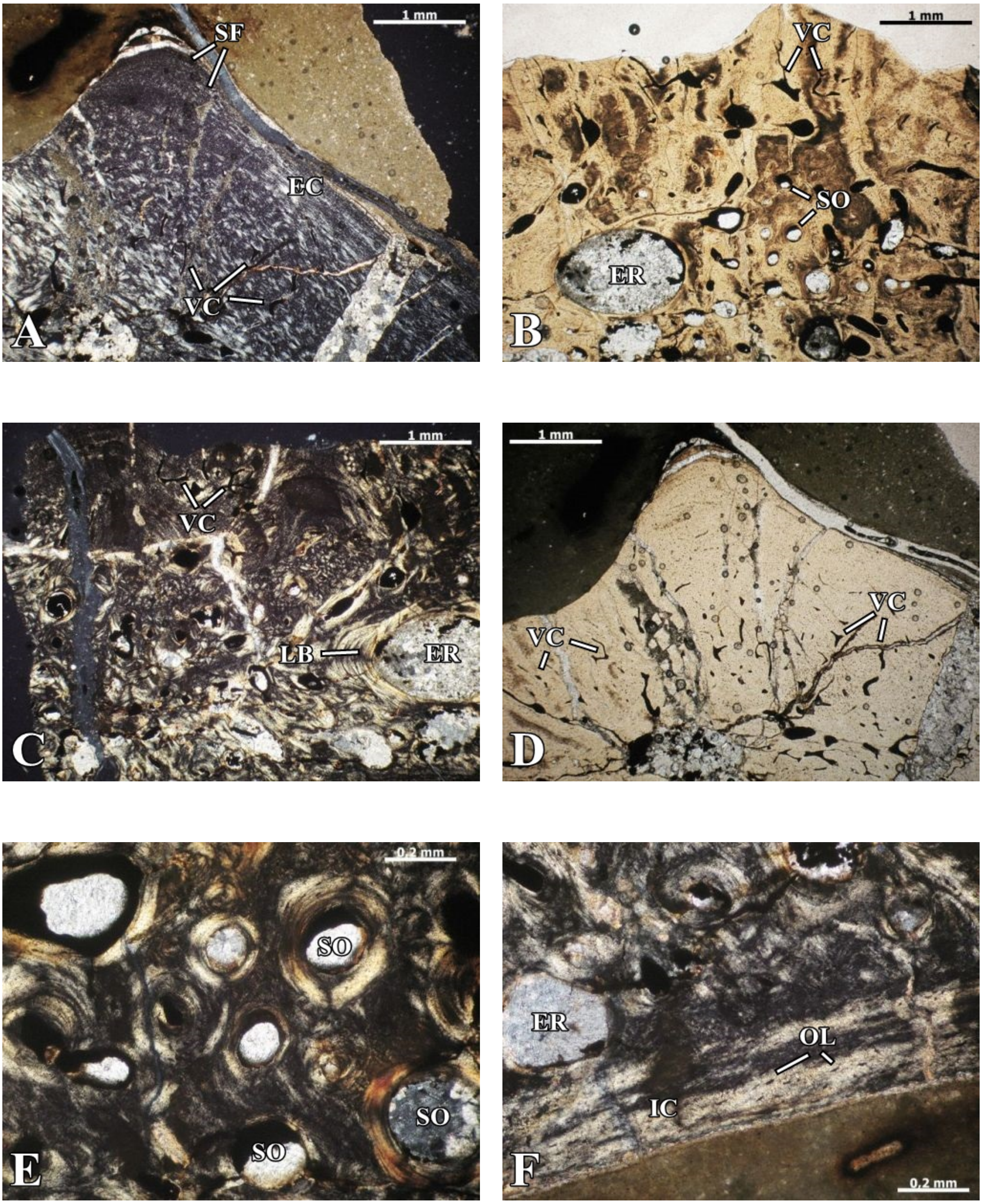

Fig. 22. The histological details of the squamosal 1 of *Metoposaurus krasiejowensis*. **(A)** Thick external cortex in the area of sculptural ridges with distinct Sharpey's fibers; image in CPL; **(B-D)** Well vascularized external cortex (vascular canals) and middle region (secondary osteons); images in PPL (B, D) and CPL (C); **(E)** Dense accumulation of large secondary osteons; image in CPL; **(F)** Avascular internal cortex with distinct osteocyte lacunae; image in CPL. Abbreviations: EC = external cortex, SF = Sharpey's fibers, VC = vascular canals, ER = erosion cavities, SO = secondary osteons, LB = lamellar bone, IC = internal cortex, OL = osteocyte lacunae, PPL = plane polarized light, CPL = cross polarized light.

Squamosal 2

The histological thin section was prepared from the posterior (near the otic notch) part of the right squamosal. The external surface of squamosal 2 is characterized by a shallow sculpture (Fig. 23). The bone thickness is about 3 mm, and the ratio E:M:I is 1:0.3:0.5. It is the only bone in which the middle region is the thinnest part of the bone.

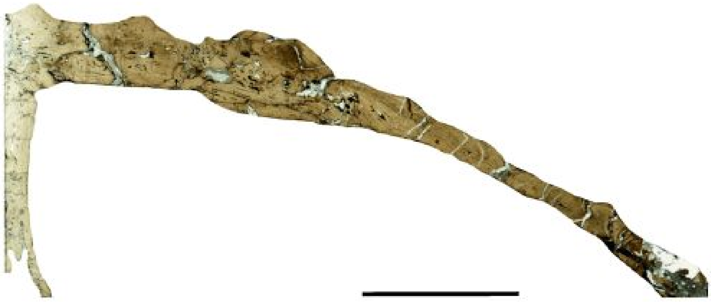

Fig. 23. Histological section of the right squamosal 2. Scale bar equals 10 mm.

External cortex - The external cortex with an average thickness of about 1100 µm, consists of parallel-fibered bone (Fig. 24A). Next to the surface the thin, avascular layer with thick collagen fibers is visible. Deeper part of the cortex is poor vascularized, mainly by the irregularly shaped vascular canals. The thin Sharpey's fibers occur in the deeper parts of the sculptural ridges (Fig. 24B). The rounded osteocyte lacunae with branched canaliculi appear in the moderate numbers (Fig. 24C). The degree of vascularization increases in the posterior part of section.

Middle region - The external cortex transfers gradually to the middle region. The middle region is poorly porous (Fig. 24D, E). Only the few erosion cavities with thick layer of lamellar bone are preserved. The entire part of middle region is highly remodeled and filled by the relatively compact secondary bone (Fig. 24F).

Internal cortex - The avascular on the entire width internal cortex consists of parallel-fibered bone. The Sharpey's fibers are not visible. The osteocyte lacunae are present in very low amounts.

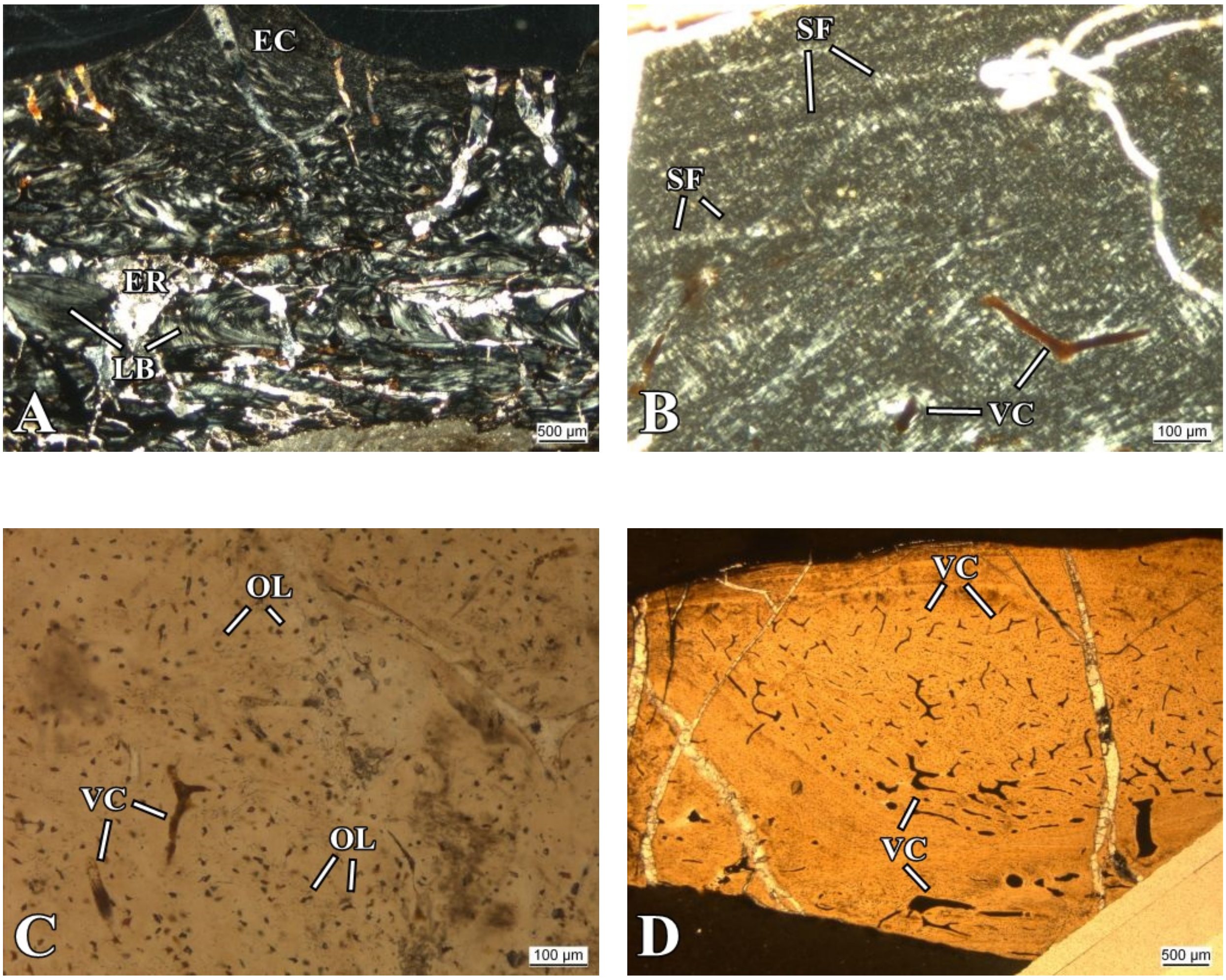

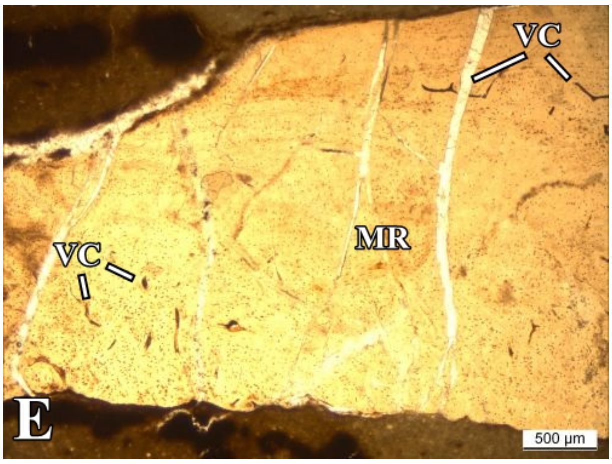

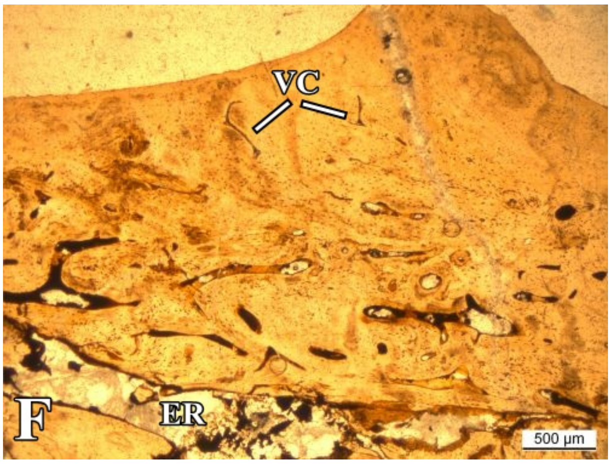

Fig. 24. The histological details of the squamosal 2 of *Metoposaurus krasiejowensis*. **(A)** Thick external cortex and highly remodeled middle region; image in CPL; **(B)** Dense bundles of thin Sharpey's fibers; image in CPL; **(C)** External cortex with visible vascular canals and rounded osteocyte lacunae; image in PPL; **(D-E)** Compact middle region with high and low degree of vascularization; images in PPL; **(F)** Poor vascularized external cortex and highly remodeled entire part of middle region; image in PPL. Abbreviations: EC = external cortex, ER = erosion cavities, LB = lamellar bone, SF = Sharpey's fibers, VC = vascular canals, OL = osteocyte lacunae, MR = middle region, PPL= plane polarized light, CPL= cross polarized light.

Postparietal

The sample was taken/prepared from the middle part of postparietal (Fig. 25). The external surface covers the ornament, about 1.5 mm high, builds up from steep ridges and polygonal pits. The sectional bone thickness is about 10 mm, and the ratio E:M:I is 1:1.9:0.5.

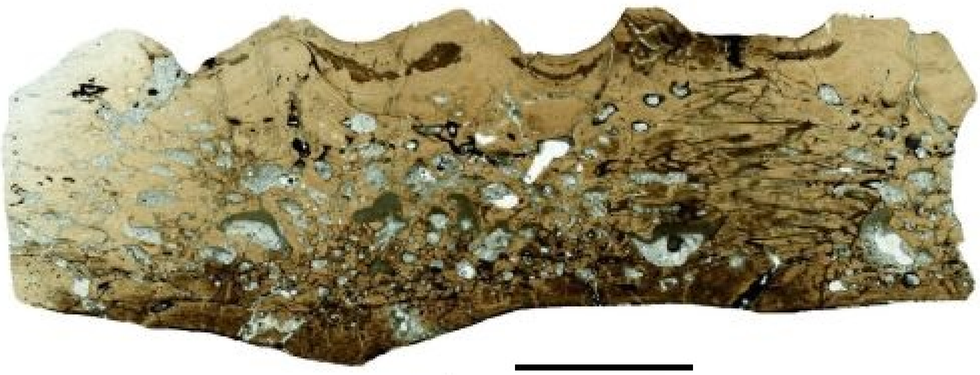

Fig. 25. Histological sections of the postparietal. Scale bar equals 5 mm.

External cortex - The external cortex of variable thickness consists of parallel-fibered bone. Its thickness is maximal in the ridges, about 2000 µm, while in the sculptural valleys is thinner having about 650 µm (Fig. 26A). The elongated simple vascular canals are present mainly in the grooves. The sculptural ridges are almost avascular. The small primary osteons, about 70 µm in diameter, appear very rarely. The thick collagen fibers form Interwoven Structural Fibers (IFS). They are usually short, but they occur on the entire width of the external cortex. The well mineralized Sharpey's fibers, with diameters about 20 µm, are numerous within the high ridges (Fig. 26B). The Sharpey's fibers occur also along the lateral edges of the cranial suture. The osteocyte lacunae with branched canaliculi are numerous in the entire external cortex. Within the sculptural ridges, the cyclical growth marks are visible as a sequence of layers of lamellar bone and layers with accumulation of ISF (Fig. 26C, D).

Middle region - The extensive and highly eroded middle region consists of the coarse cancellous and well vascularized bone. The irregular erosion cavities vary in sizes, with some up to 800 µm in diameter (Fig. 26E). Their edges are partially surrounded by approximately 120 µm thin layers of the lamellar bone. The elongated simple vascular canals are rare. The secondary osteons, with diameters ranging from 50 µm to 200 µm, are more numerous (Fig. 26F). The osteocyte lacunae, about 20 µm in diameter and with branched canaliculi occur numerously in the middle region and within the lamellar bone which surround the osteons and the erosion cavities.

Internal cortex - The internal cortex consists of parallel-fibered bone of constant thickness approximately 200 µm. The simple vascular canals are small and they occur in the whole internal cortex. The primary and secondary osteons cannot be observed. The elongated osteocyte lacunae with preserved canaliculi appear in similar quantities as in the external cortex. The Sharpey's fibers are not visible. The growth marks are visible as an alternation of lamellar layers and three resting lines.

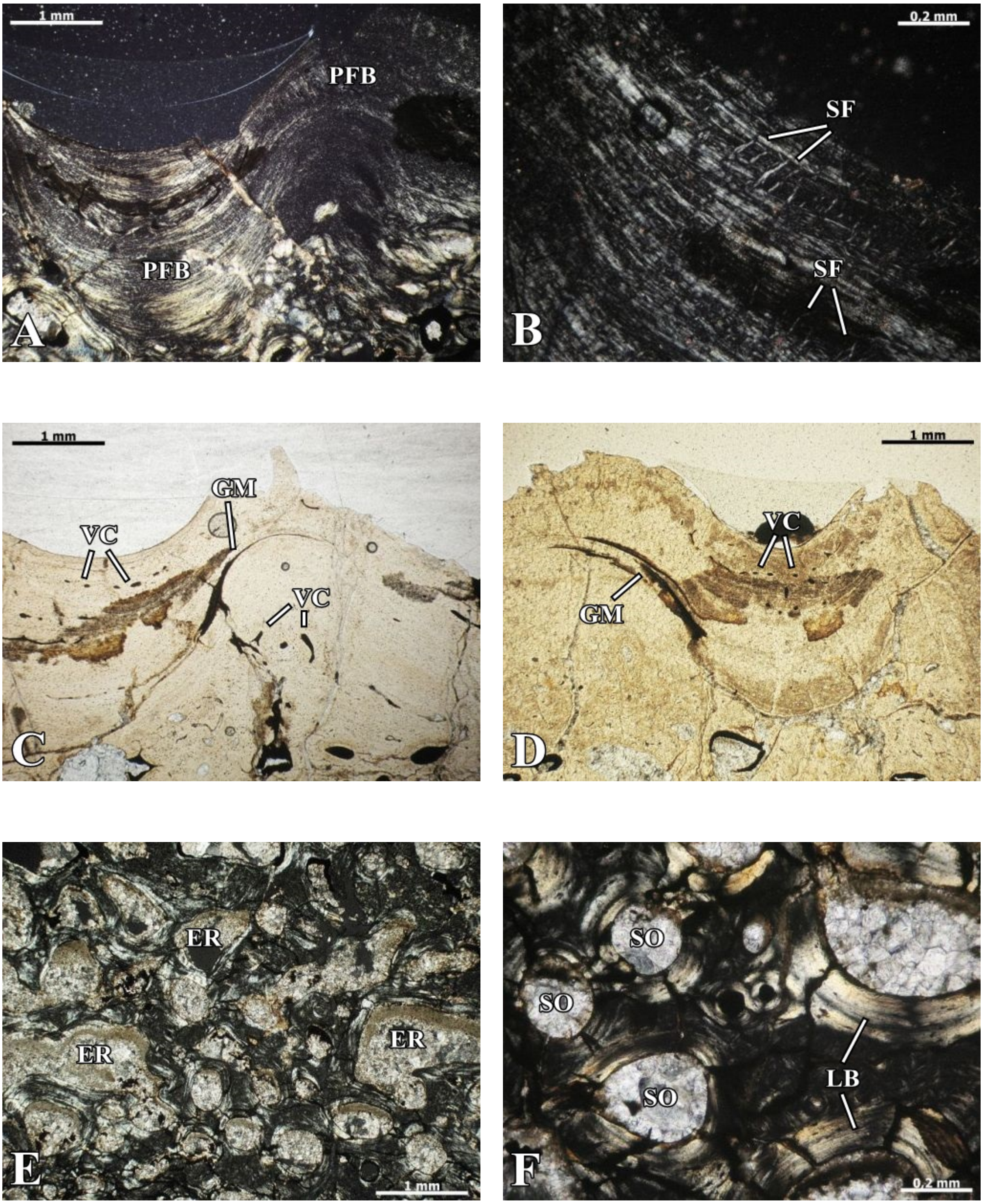

Fig. 26. The histological details of the postparietal of *Metoposaurus krasiejowensis*. **(A)** Thick layer of parallel-fibered bone in the area of sculptural vallyes and ridges; image in CPL; **(B)** Dense clumps of well mineralized Sharpey's fibers; image in CPL; **(C-D)** Distinct growth marks structure and vascular canals within the ornamented ridges; images in PPL; **(E)** Heavy eroded middle region; image in CPL; **(F)** Numerous and large

secondary osteons in the central parts of middle region; image in CPL. Abbreviations: PFB = parallel-fibered bone, SF = Sharpey's fibers, VC = vascular canals, GM = growth marks structure, ER = erosion cavities, SO = secondary osteons, LB = lamellar bone, PPL = plane polarized light, CPL = cross polarized light.

Tabular

The histological sample was prepared from the right tabular, from about the middle part of this bone (Fig. 27). The external surface of tabular bears the polygonal sculpture consists of high, about 1 mm ridges and wide pits. The bone thickness ranges from 10 mm to 12 mm, and the ratio E:M:I is 1:2.2:0.8.

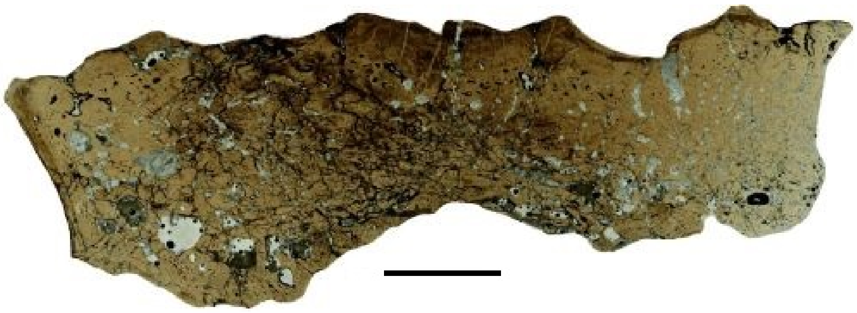

Fig. 27. Histological section of the right tabular. Scale bar equals 5 mm.

External cortex - The about 1000 µm thick well vascularized external cortex consists of parallel-fibered bone (Fig. 28A). The numerous and irregular in shape simple vascular canals mainly occur in the central and deeper parts of external cortex. The simple vascular canals, about 350 µm in length, are the most numerous within the sculptural ridges, while in the valleys their length does not exceed 150 µm (Fig. 28B). They are arranged in rows, along to superficial edge of the bone. The primary osteons are small, about 50 µm in diameter, and they occur in the central parts of the external cortex and in the border with the middle region. The numerous and well mineralized Sharpey's fibers, with a diameter about 20 µm, occur in the upper and deeper parts of the cortex (Fig. 28C). The distinct collagen fibers create a thick Interwoven Structural Fibers (IFS). Within the sculptural ridges, the cyclical growth marks,

manifested as a sequence of thin resting lines and the layer of parallel-fibered bone are present (Fig. 28D). In some places the alternation of valleys and ridges is preserved. The osteocyte lacunae with branched canaliculi and rounded in shape occur numerously within the bone matrix.

Middle region - The middle region consists of coarse cancellous bone. The very large erosion cavities, with a diameter up to 1500 µm, occur only in the deeper parts of the middle region, partially in the border with the internal cortex. Their edges are fragmentary surrounded by a thin layer of the lamellar bone. The most part of the middle region is highly remodeled; however the porosity of this bone is low (Fig. 28E). The secondary osteons, with a diameter about 120 µm, are more numerous. The slightly elongated osteocyte lacunae, about 10 µm in length, and with branched canaliculi are common in the middle region.

Internal cortex - The internal cortex with constant thickness of about 1000 µm, consists of parallel-fibered bone. The irregularly in shapes, simple vascular canals, about 100 µm in length, are present in large amounts (Fig. 28F). In some areas they are arranged in several rows at angle of about 40^0 to the internal cortex. The secondary osteons rarely appear, and in some places they penetrate into the internal cortex from the middle region. The numerous Sharpey's fibers are packed in bundles. The elongated osteocyte lacunae, about 10 µm in length and with branched canaliculi are numerous.

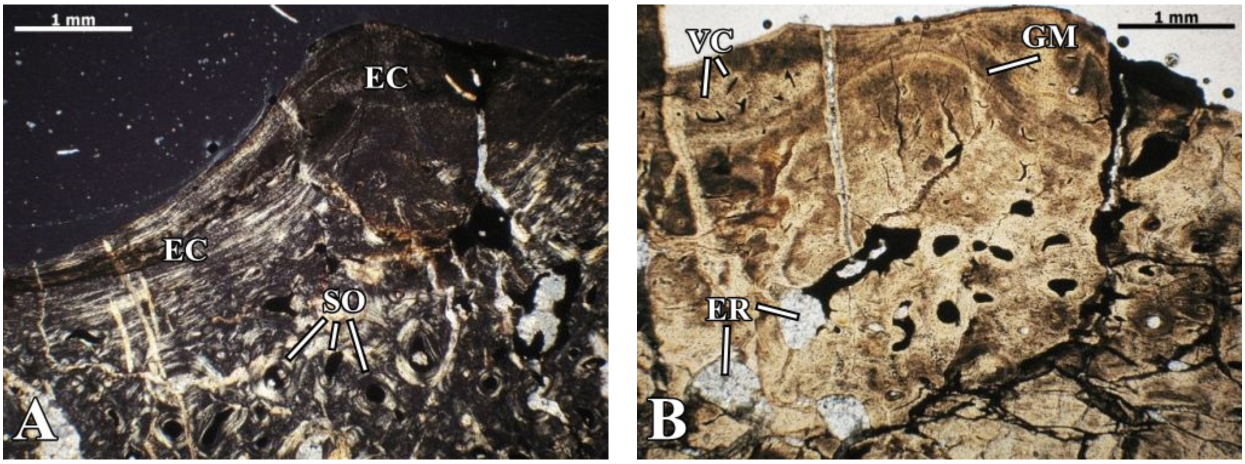

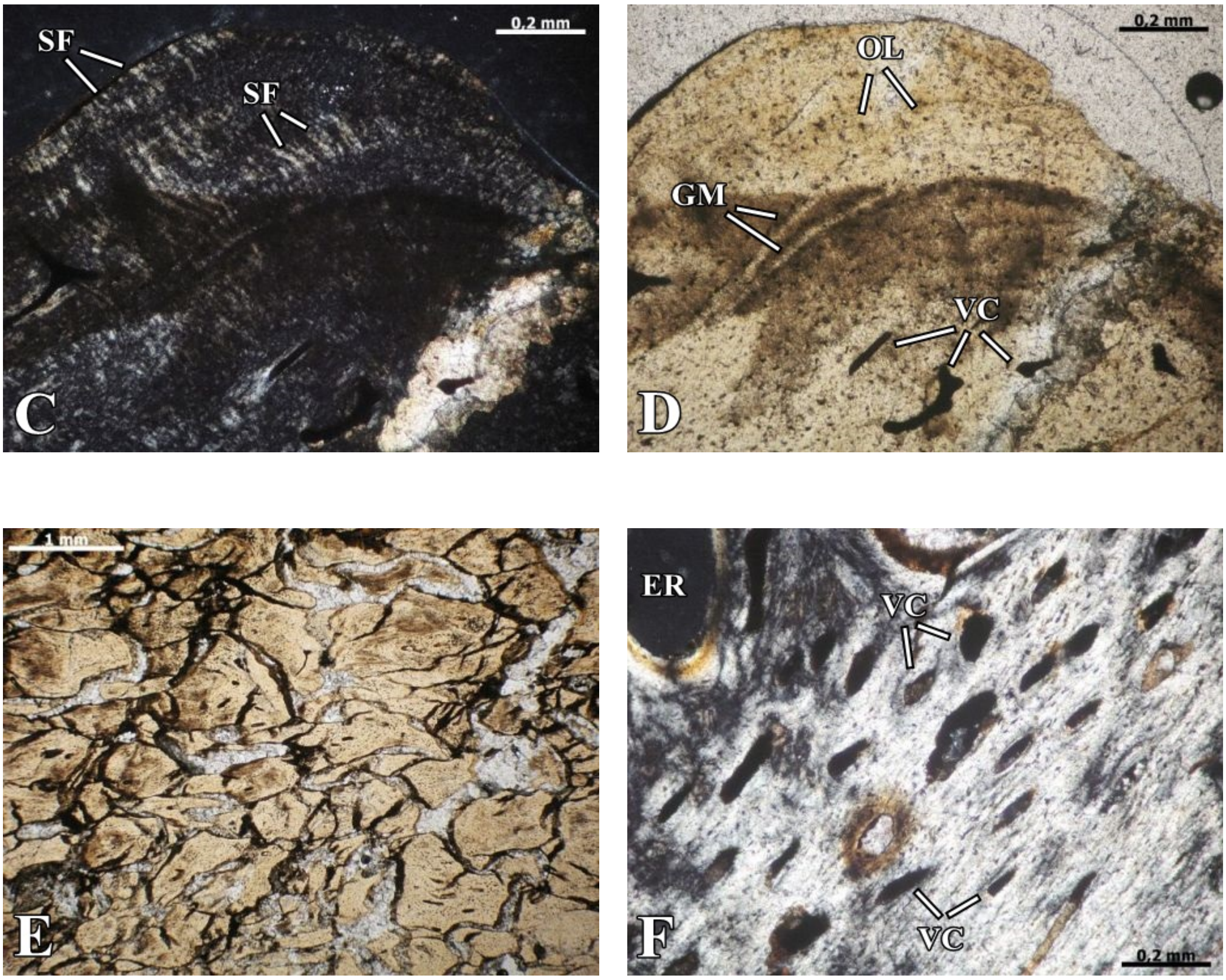

Fig. 28. The histological details of the tabular of *Metoposaurus krasiejowensis*. **(A-B)** External cortex and middle region; images in CPL (A) and PPL (B); **(C)** Numerous and packed in dense bundles Sharpey's fibers visible in the external cortex; image in CPL; **(D)** Growth marks structure in the area of sculptural ridges; image in PPL; **(E)** Heavy eroded middle region; image in PPL; **(F)** Numerous vascular canals in the parallel-fibered bone of internal cortex; image in CPL. Abbreviations: EC = external cortex, SO = secondary osteons, ER = erosion cavities, VC = vascular canals, SF = Sharpey's fibers, GM = growth marks structure, OL = osteocyte lacunae, PPL = plane polarized light, CPL = cross polarized light.

Quadratojugal

The histological sample was prepared from the middle part of the left quadratojugal. The external surface of the bone possesses distinct sculpture, which consists of about 1 mm

high ridges and the deep grooves (Fig. 29). The average thickness of quadratojugal is 5 mm, and the ratio E:M:I is 1:1.7:0.6.

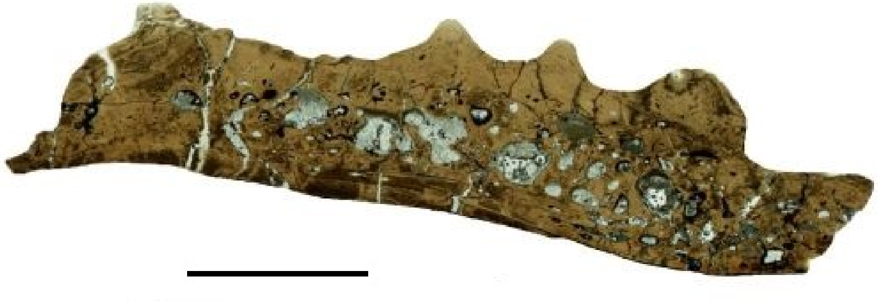

Fig. 29. Histological section of the left quadratojugal. Scale bar equals 5 mm.

External cortex - The external cortex consists of parallel-fibered bone of a variable thickness. The thickest bone, exceeding 1000 µm, is in the area of the sculptural grooves (Fig. 30A). Various in sizes and shape simple vascular canals are numerous, especially within the ridges (Fig. 30B). The small primary osteons are rare. The well mineralized Sharpey's fibers can be observed within the sculptural ridges and in the ridges (Fig. 30C). The cyclical growth marks, manifested as a sequence of thin resting lines and layers of parallel-fibered bone, are present within the sculptural ridges. In some places the alternation of valleys and ridges is preserved. The osteocyte lacunae, about 10 µm in diameter and with branched canaliculi are numerous.

Middle region - The large and well vascularized middle region consists of cancellous bone. The large erosion cavities, about 1500 µm in diameter, occur in the central part of the middle region. The small and irregular simple vascular canals are not as numerous as in the external cortex. The secondary osteons varying in sizes appear in the larger amount. The largest of them reached almost 200 µm in diameter. The small secondary osteons, with a diameter about 40 µm, form the Haversian tissue in some parts of the middle region (Fig. 30D, E). The mostly rounded osteocyte lacunae with long, branched canaliculi are numerous.

Internal cortex - The relatively thick, of about 700 μm, and well vascularized internal cortex consists of parallel-fibered bone. The irregular and highly elongated, up to 300 μm long, simple vascular canals are numerous. Sometimes they are arranged in several, regular rows, parallel to cortex fibers (Fig. 30F). The secondary osteons of the average size appear in a much lower amount. The Sharpey's fibers are not visible. The rounded osteocyte lacunae with preserved canaliculi are very numerous.

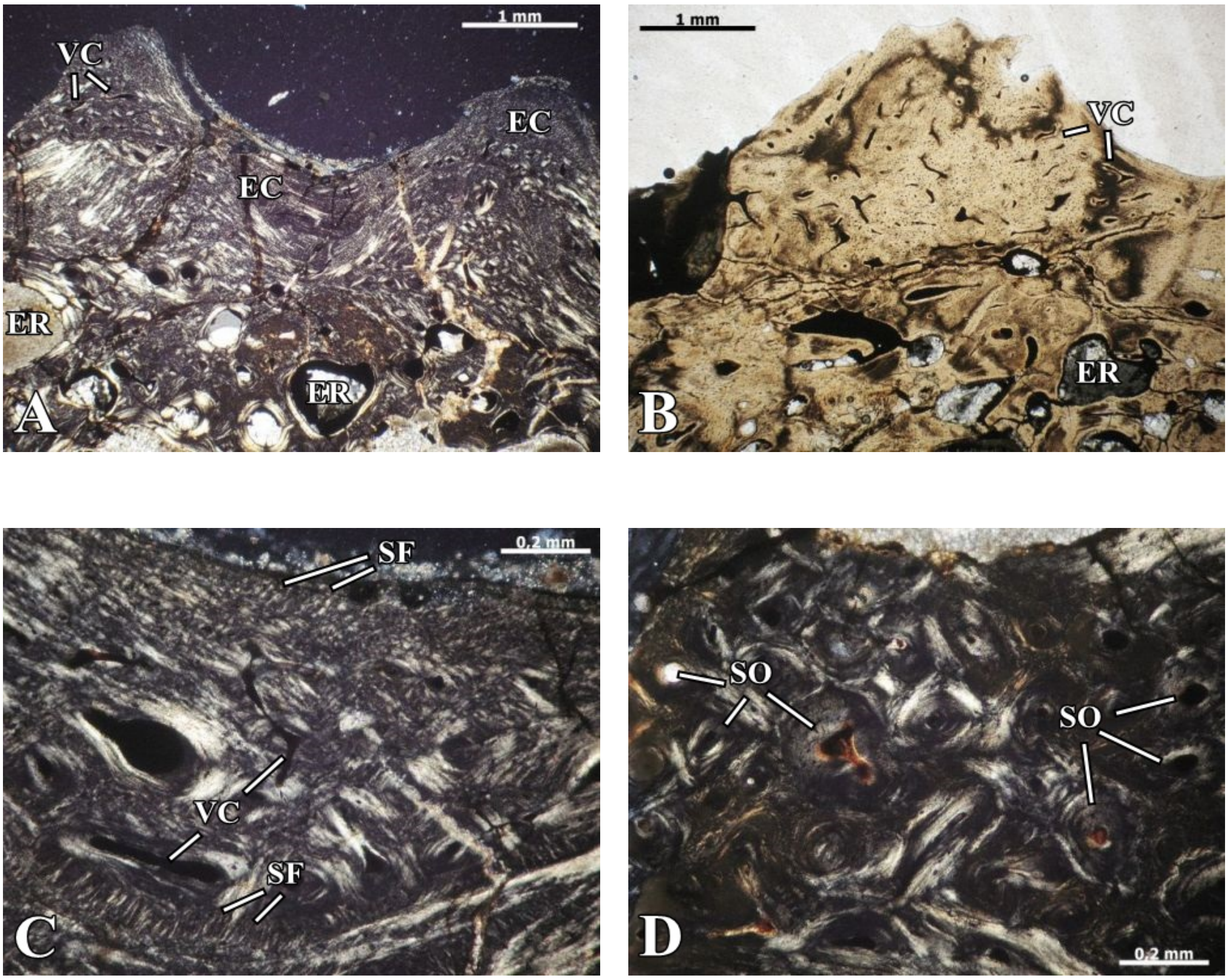

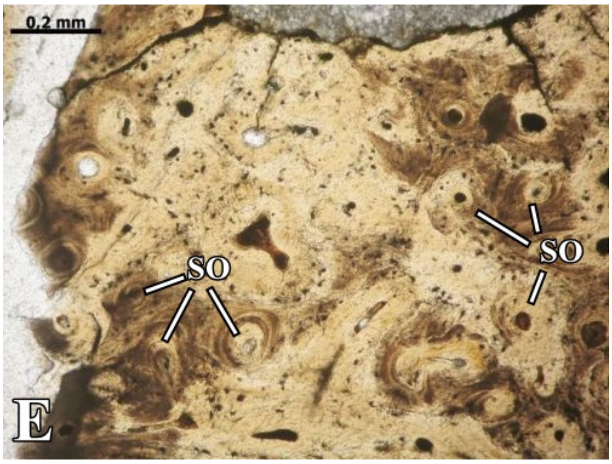

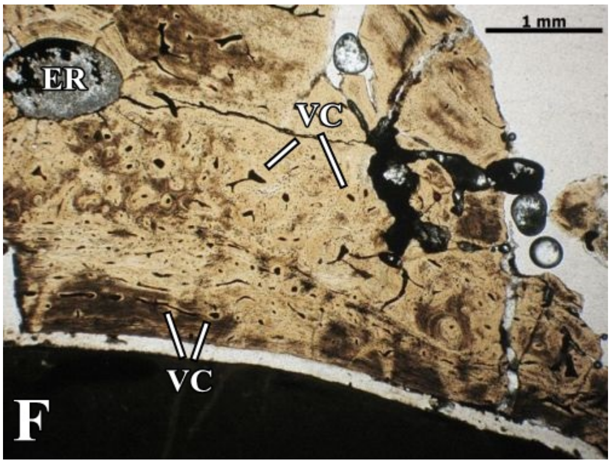

Fig. 30. The histological details of the quadratojugal of *Metoposaurus krasiejowensis*. **(A)** External cortex and middle region; image in CPL; **(B)** Numerous vascular canals in the area of sculptural ridges; image in PPL; **(C)** Well mineralized bundles of Sharpey's fibers in external cortex; image in CPL; **(D-E)** Incipient Haversian tissue; images in CPL (D) and PPL (E); **(F)** Well vascularized part of internal cortex; image in PPL. Abbreviations: EC = external cortex, ER = erosion cavities, VC = vascular canals, SF = Sharpey's fibers, SO = secondary osteons, PPL = plane polarized light, CPL = cross polarized light.

Histology of the dermal bones of the palate

Vomer

The thin section examined histologically was prepared from the left part of the vomer (Fig. 31). The external surface of the bone bears a small, about 150 μm in high circum-vomerine teeth. The sectional bone thickness varies from 3 mm to 6 mm, and the ratio E:M:I is 1:2.8:0.7.

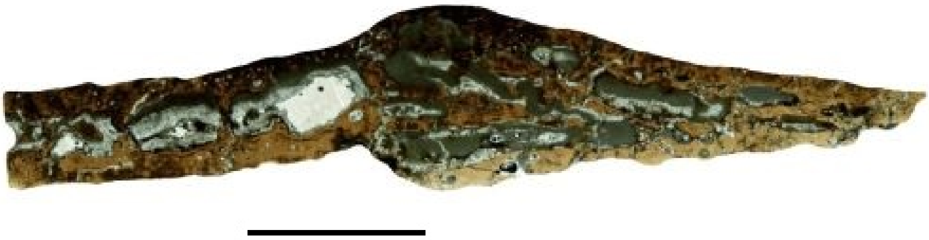

Fig. 31. Histological section of the vomer. Scale bar equals 5 mm.

External cortex - The well vascularized external cortex with maximal thickness about 450 µm, consists of parallel-fibered bone (Fig. 32A). The secondary osteons with diameters of 70 µm are numerous. The simple vascular canals appear in a lower amount. The Sharpey's fibers are very rare. The elliptical osteocyte lacunae of approximately 20 µm in length are arranged with their long axis parallel to the collagen fibers.

Middle region - The large and highly eroded middle region consists of cancellous bone. The erosion cavities reach the largest size among all of the dermal bone examined (Fig. 32B, C). They are even 3000 µm in length and are partially surrounded by a thin layer of the lamellar bone. The secondary osteons, of about 70 µm in diameter, occur mainly in the subsurface and in the deeper parts of the middle region. The rounded osteocyte lacunae are numerous and possess branching canaliculi.

Internal cortex - The internal cortex with a constant thickness of approximately 650 µm, consists of parallel-fibered bone. The very large secondary osteons, about 70 µm in diameter, are numerous and in some areas are arranged in orderly rows (Fig. 32D & 32E). The simple vascular canals are not visible. The well mineralized Sharpey's fibers occur in the subsurface parts of the internal cortex and are oriented at an angle of 45^0 to the bone surface (Fig. 32F). The rounded osteocyte lacunae with a diameter of about 10 µm and with branched canaliculi are very numerous.

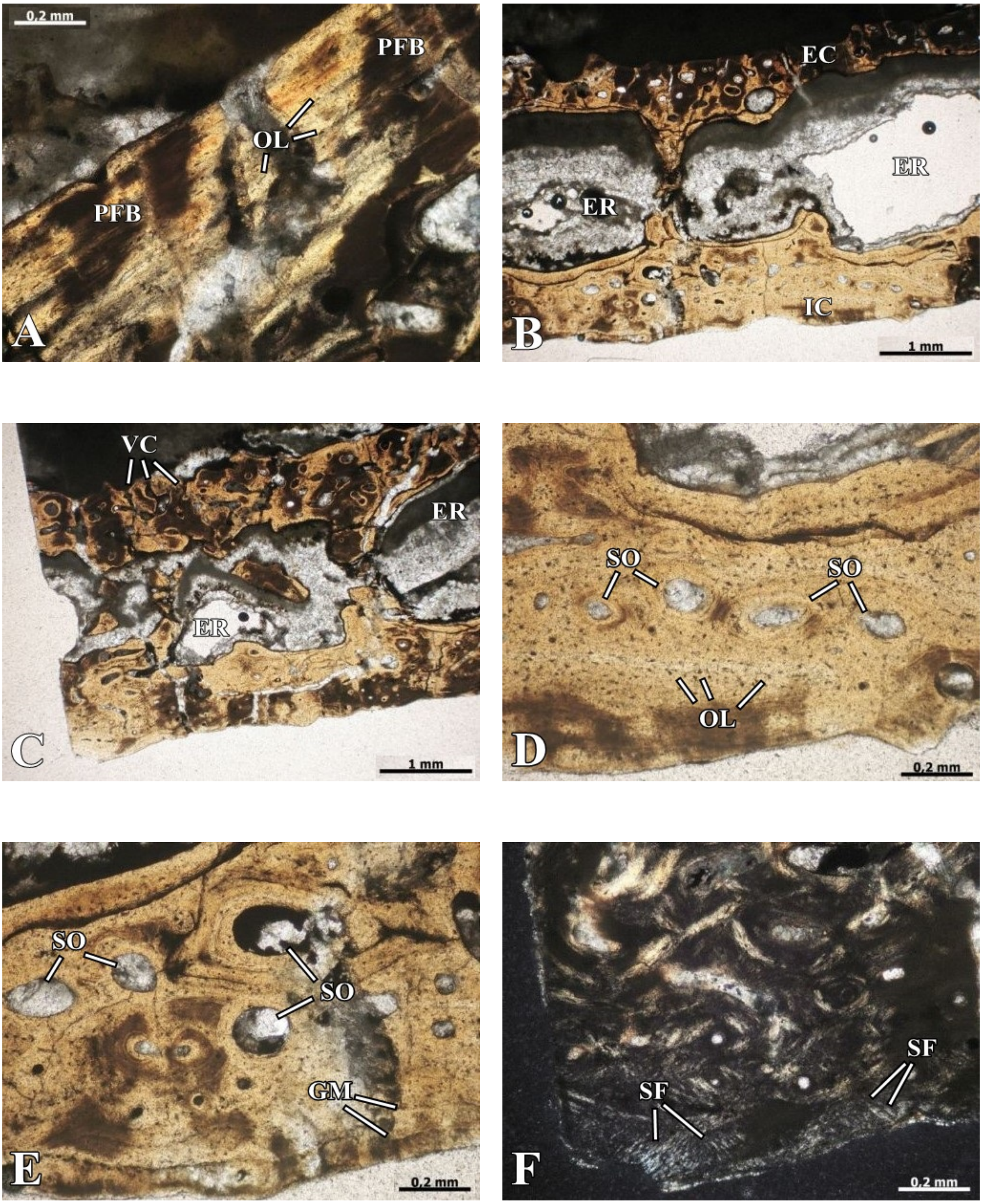

Fig. 32. The histological details of the vomer of *Metoposaurus krasiejowensis*. **(A)** External cortex in cross polarized light; **(B-C)** Large erosion cavities within the middle region; images in PPL; **(D-E)** Internal cortex with well develop vascular network and with distinct growth marks structures; images in PPL; **(F)** Numerous Sharpey's fibers in the upper part of internal cortex; image in CPL. Abbreviations: SF = Sharpey's fibers, PO = primary osteons, SO = secondary osteons, GM = growth marks structure, ER = erosion cavities, VC = vascular

canals, IC = internal cortex, PFB = parallel-fibered bone, OL = osteocyte lacunae, PPL = plane polarized light, CPL = cross polarized light.

Parasphenoid

The thin section was made from the posterior part of *processus cultriformis* (Fig. 33). The bone has the clearly ornamented external surface (located ventrally). The sectional bone thickness ranges from 2 mm to 5 mm, and the ratio E:M:I is 1:2:1.5.

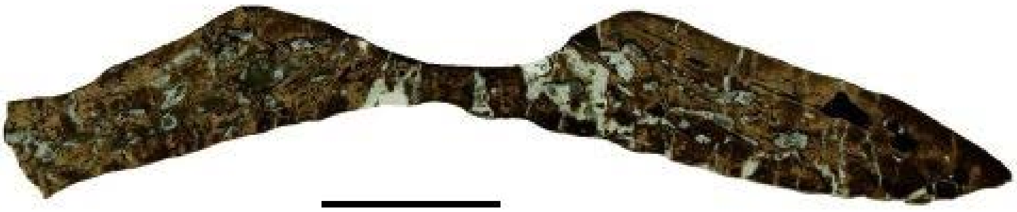

Fig. 33. Histological section of the parasphenoid. Scale bar equals 5 mm.

External cortex - The external cortex consists of parallel-fibered bone and it has a constant thickness, of an average of 900 µm. In the deeper parts of the external cortex, the highly elongated simple vascular canals are arranged in several rows parallel to the cortex surface (Fig. 34A). The secondary osteons appear in a very small amount. The Sharpey’s fibers are not visible. The elongated osteocyte lacunae, 10 µm in diameters, are very numerous and have branched canaliculi.

Middle region - The coarse cancellous middle region is well vascularized. The numerous erosion cavities have various sizes, ranging from 300 µm to over 2500 µm in length (Fig. 34A & 34B). Their lateral edges are partially surrounded by thin layers of the lamellar bone. The middle region possess well-developed vascular network. The secondary osteons, with diameters ranging from 80 µm to nearly 200 µm, appear in a large amount, and they are lined with thick, up to 100 µm, layer of the lamellar bone (Fig. 34C). The simple vascular

canals are not visible within the middle region. The osteocyte lacunae with a diameter 10 μm, and with branched canaliculi are numerous (Fig. 34D).

Internal cortex - The relatively thin and moderate vascularized internal cortex consists of parallel-fibered bone. The irregular in shape, small simple vascular canals and secondary osteons, about 60 μm in diameter, are not numerous. The Sharpey's fibers are absent. The numerous, rounded and elongated osteocyte lacunae have branched canaliculi. The alternation of two thick well vascularized and two avascular layers is visible, represent the zones and annuli, respectively.

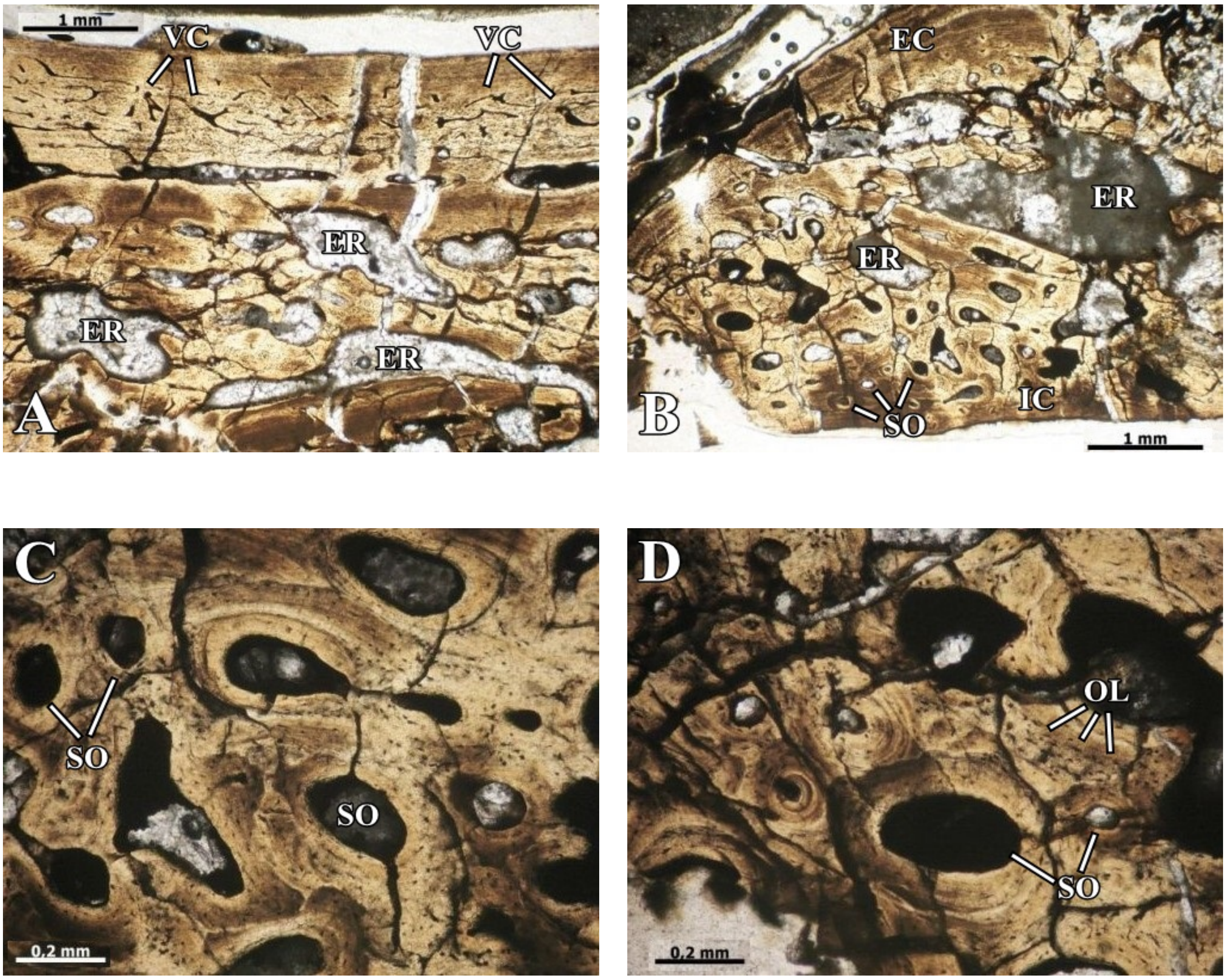

Fig. 34. The histological details of the parasphenoid of *Metoposaurus krasiejowensis*. **(A)** Cross section of the parasphenoid with clearly visible well vascularized external cortex and remodeled middle region; image in PPL; **(B)** Heavy eroded middle region; image in PPL; **(C-D)** Large and numerous secondary osteons in middle region;

images in PPL. Abbreviations: EC = external cortex, SO = secondary osteons, ER = erosion cavities, VC = vascular canals, IC = internal cortex, OL = osteocyte lacunae, PPL = plane polarized light, CPL = cross polarized light.

Pterygoid

The histological section was prepared from the posterior part of the palatine branch, of the right pterygoid, in a place where the bone is thicker and contacts with the parasphenoid (Fig. 35). The external part of pterygoid is poorly preserved, but the preserved areas show a lack of the ornamentation. The average bone thickness is approximately 8 mm, and the ratio E:M:I is 1:5.4:2.

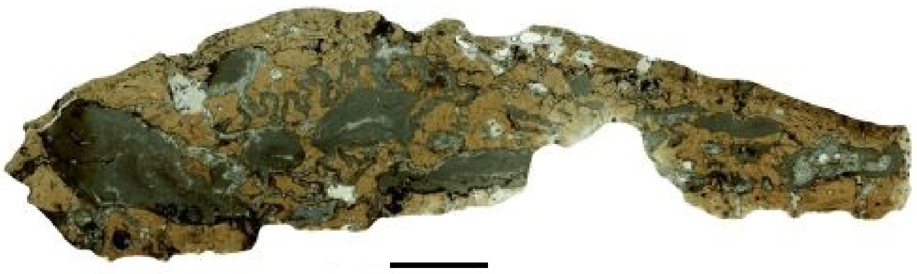

Fig. 35. Histological section of the right pterygoid. Scale bar equals 5 mm.

External cortex - The well vascularized external cortex, about 550 µm thick, consists of parallel-fibered bone. The numerous and elongated simple vascular canals are arranged parallel to the external cortex. The small secondary osteons, with a diameter about 60 µm, appear in smaller amounts. The Sharpey's fibers are not observed. The numerous osteocyte lacunae are variable in shapes.

Middle region - The coarse cancellous middle region is highly eroded. The irregularly shaped erosion cavities are very large. Their size varies from 500 µm to over 3500 µm, and their edges are surrounded by thick, about 200 µm, layer of the lamellar bone (Fig. 36A, B). The nutritional canals, 180 µm in diameter, occur in the middle region. Because the middle

region is highly eroded, so the simple vascular canals and osteons are not visible. The secondary osteons, with a diameter about 100 μm, occur only in the subsurface parts and on the border with the internal cortex. The osteocyte lacunae, about 20 μm in diameter, are very numerous.

Internal cortex - The partially preserved internal cortex consists of parallel-fibered bone. Its thickness is about 550 μm. The simple vascular canals are absent and the small secondary osteons, with diameters of 70 μm, occur very rarely. The Sharpey's fibers are not visible. However, the elliptically osteocyte lacunae are numerous and they are parallel to the internal cortex.

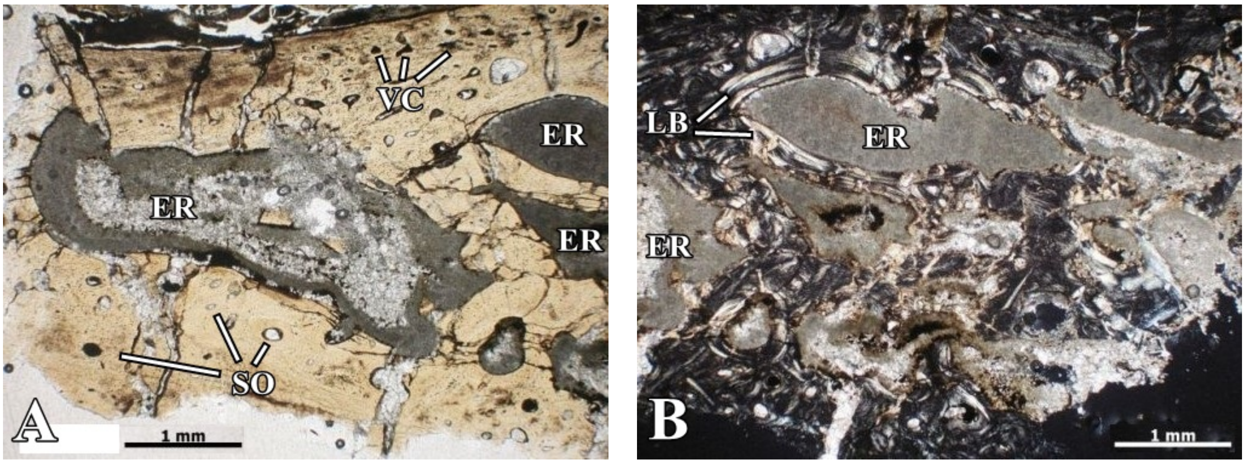

Fig. 36. The histological details of the pterygoid of *Metoposaurus krasiejowensis*. **(A-B)** Cross section from the pterygoid in PPL (A) and CPL (B) with distinct large and irregular erosion cavities. Abbreviations: SO = secondary osteons, ER = erosion cavities, VC = vascular canals, LB = lamellar bone, PPL = plane polarized light, CPL = cross polarized light.

Quadrate

The left quadrate was sectioned transversally in the central part of the bone (Fig. 37). The diameter of the quadrate is about 20 mm.

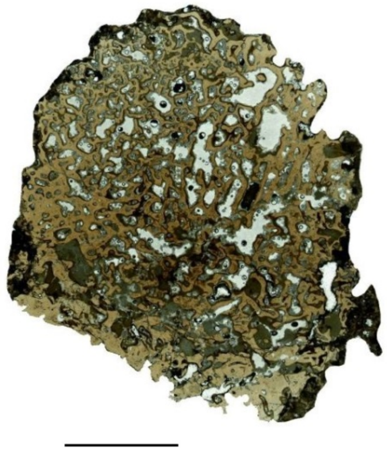

Fig. 37. Histological section of the left quadrate. Scale bar equals 5 mm.

The partially preserved and well vascularized cortex consists of parallel-fibered bone. Its thickness is 200 µm. The simple vascular canals occur sporadically, but the secondary osteons, with diameters ranging from 60 µm to 170 µm, are more numerous (Fig. 38A). The lamellar bone inside the secondary osteons is relatively thick, up to 60 µm. The Sharpey's fibers are very short and they occur only in the subsurface parts of the cortex. The elongated osteocyte lacunae mainly are present within the lamellar bone. They not possess canaliculi. The growth marks are not visible.

The central part of the bone is trabecular with irregular and variable in thickness and length bone trabeculae. The trabecular thickness is approximately 150 µm. The most numerous trabeculae are in the central parts of the bone, less frequent, shorter and thicker, on the border with cortex (Fig. 38B, C). On the border with cortex the secondary osteons, with a diameter of about 100 µm, are observed. The elongated osteocyte lacunae to about 20 µm, in the subsurface parts of the cancellous bone are rare, while in the deeper parts of the middle region their number rapidly increasing. In the central part of middle region, the relatively large accumulations of calcified cartilage remains are visible (Fig. 38D).

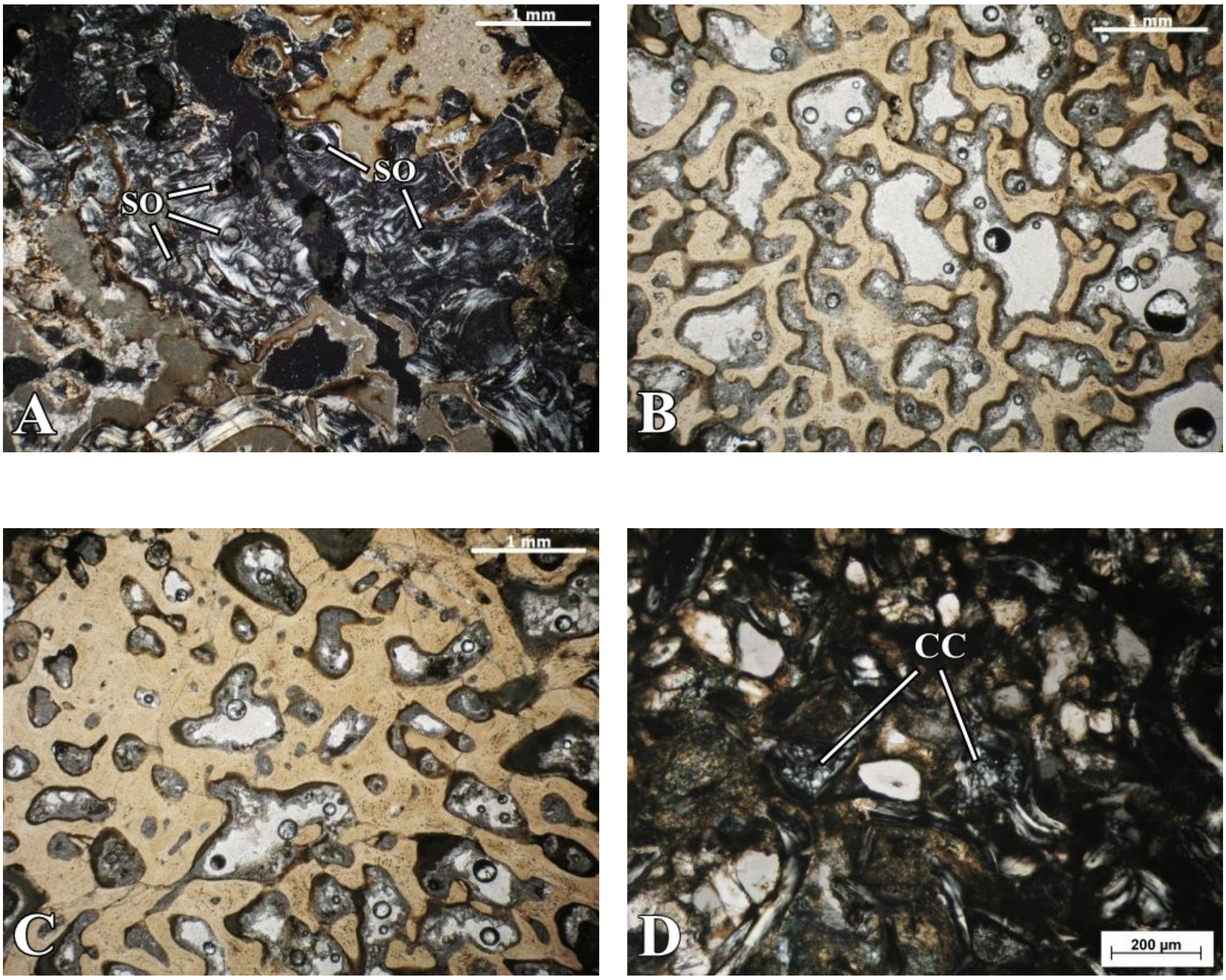

Fig. 38. The histological details of the quadrate of *Metoposaurus krasiejowensis*. **(A)** External surface of quadrate; image in CPL; **(B-C)** Trabecular middle region; images in PPL; **(D)** Middle region with distinct remains of calcified cartilage; image in CPL. Abbreviations: SO = secondary osteons, CC = calcified cartilage, PPL = plane polarized light, CPL = cross polarized light.

Exoccipital

The thin section was prepared from the right exoccipital. The bone was cut approximately in their central part (Fig. 39). The diameter of the bone is about 20 mm.

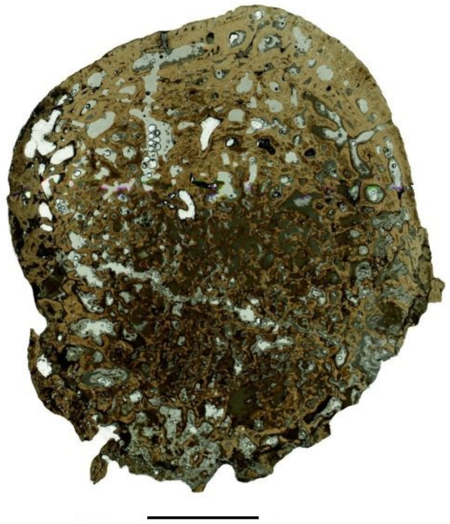

Fig. 39. Histological section of the right exoccipital. Scale bar equals 5 mm.

The cortex, with a thickness of more than 2000 µm, consists of parallel-fibered bone. The entire cortex is relatively well vascularized. The simple vascular canals occur rarely, secondary osteons are relatively numerous. The single rounded erosion cavities, with diameters of about 900 µm are visible. The well mineralized, very numerous and packed in densely bundles Sharpey’s fibers are present (Fig. 40A, B). They occur in the entire cortex, and the diameter of some bundles can reach up to 50 µm. The rounded osteocyte lacunae are numerous in the cortex. The growth marks are absent.

The central part of the bone consists of an irregular network of bony trabeculae, with large pore spaces between them. The number and length of trabeculae increase in the central area of bone. The thickness of the trabeculae is small in these areas, to about 200 µm (Fig. 40C, D). In the subsurface parts of the middle region, the trabeculae thickness can rise almost double. The elongated osteocyte lacunae, with a diameter of about 10 µm, are very numerous and irregularly arranged.

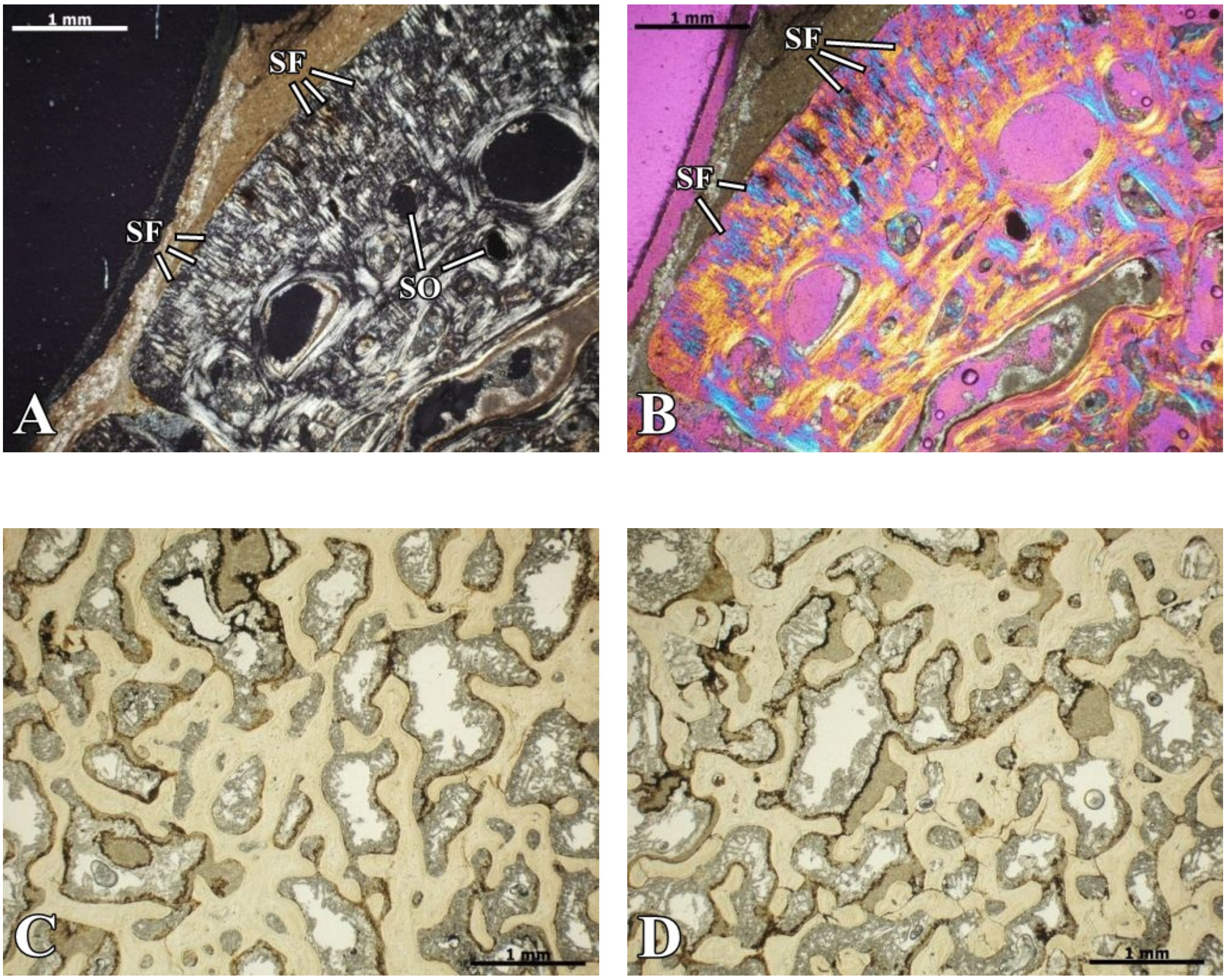

Fig. 40. The histological details of the exoccipital of *Metoposaurus krasiejowensis*. **(A-B)** External surface of exoccipital, with distinct thick and numerous Sharpey's fibers; images in CPL (A) and in ¼ Lambda filter (B); **(C-D)** Middle region with dense network of bony trabeculae; images in PPL; images in CPL. Abbreviations: SF = Sharpey's fibers, SO = secondary osteons, , PPL = plane polarized light, CPL = cross polarized light.
